# Exploiting E3 ubiquitin ligases to reeducate the tumor microenvironment for cancer therapy

**DOI:** 10.1186/s40164-023-00394-2

**Published:** 2023-03-30

**Authors:** Xian-Miao Li, Zhen-Yu Zhao, Xiao Yu, Qi-Dong Xia, Peng Zhou, Shao-Gang Wang, Huan-Lei Wu, Jia Hu

**Affiliations:** 1 Department of Urology, Institute of Urology, Tongji Hospital, Tongji Medical College, Huazhong University of Science and Technology, Liberalization Ave, No. 1095, Wuhan, 430030 China; 2 Department of Geriatrics, Tongji Hospital, Tongji Medical College, Huazhong University of Science and Technology, Wuhan, 430030 China

**Keywords:** Tumor microenvironment, Ubiquitination, Deubiquitinases, Cancer therapy, SUMOylation

## Abstract

Tumor development relies on a complex and aberrant tissue environment in which cancer cells receive the necessary nutrients for growth, survive through immune escape, and acquire mesenchymal properties that mediate invasion and metastasis. Stromal cells and soluble mediators in the tumor microenvironment (TME) exhibit characteristic anti-inflammatory and protumorigenic activities. Ubiquitination, which is an essential and reversible posttranscriptional modification, plays a vital role in modulating the stability, activity and localization of modified proteins through an enzymatic cascade. This review was motivated by accumulating evidence that a series of E3 ligases and deubiquitinases (DUBs) finely target multiple signaling pathways, transcription factors and key enzymes to govern the functions of almost all components of the TME. In this review, we systematically summarize the key substrate proteins involved in the formation of the TME and the E3 ligases and DUBs that recognize these proteins. In addition, several promising techniques for targeted protein degradation by hijacking the intracellular E3 ubiquitin-ligase machinery are introduced.

## Background

The tumor microenvironment (TME) refers to a complex ectopic ecosystem composed of multiple cell types (immune cells, fibroblasts, endothelial cells), as well as extracellular components, including extracellular matrix (ECM) and secreted mediators (growth factors and cytokines, chemokines) [1]. Bidirectional communication between tumor cells and the TME is not only directly responsible for the genesis, progression and metastasis of tumors but is also closely related to the therapeutic efficacy and long-term prognosis of patients [2–4]. Although nonmalignant stromal cells in the TME have the same origin as adjacent normal tissue cells, they can be hijacked by tumor cells to create a protumorigenic microenvironment along with tumor cells. Various signaling pathways, transcription factors, and key enzymes are involved in adjusting the TME, which is a highly complex network.

**Fig. 1:**
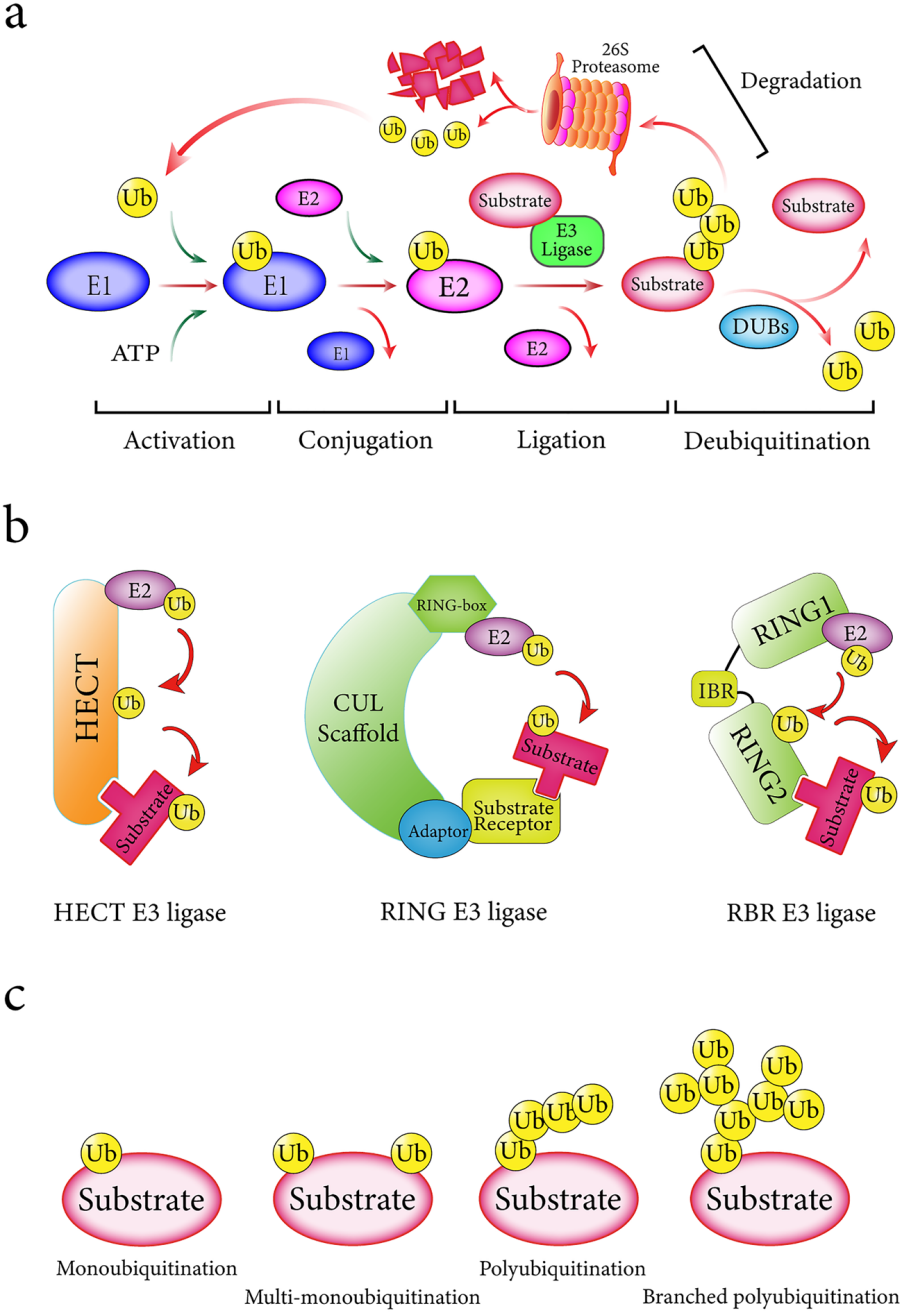
The ubiquitination process and ubiquitin code. **a**. The cascade of ubiquitin modification. Ubiquitin modification involves three successive steps catalyzed by three types of enzymes: (1) E1 ubiquitin-activating enzyme activates ubiquitin in an ATP-dependent manner; (2) the activated ubiquitin protein is transferred to E2 ubiquitin- conjugating enzyme via a transesterification reaction; (3) E3 ubiquitin ligase bridges the ubiquitin-loaded E2 and targeted protein to facilitate the binding of ubiquitin with a substrate lysine. These attached ubiquitin molecules can be cleaved by deubiquitinases from substrates. Polyubiquitin chains represented by the K48 linkage will lead to 26S proteasome‑mediated proteolysis. **b**. Differential catalytic domains and transfer modes of three classes of E3 ligases. **c**. Topological versatility in protein ubiquitination

Ubiquitination is the most common posttranscriptional modification after phosphorylation, in which 76-amino-acid ubiquitin molecules are covalently conjugated to the substrate protein [5, 6]. Ubiquitin (Ub), which is a highly conserved modifier, is attached to substrate proteins by a cascade of three enzymes: E1 Ub-activating enzyme, E2 Ub-conjugating enzyme, and E3 ubiquitin ligase [7]. First, E1 activates ubiquitin in an ATP-dependent manner and then transfers it to E2 via a transesterification reaction. Finally, E3 bridges the Ub-loaded E2 and target protein to facilitate the binding of ubiquitin with a substrate lysine (K), resulting in protein ubiquitination. E2 ligases are more than just carriers, showing specificity in E2-E3 pairings and regulating the progressivity and topology of ubiquitin chain assembly [8, 9]. Ubiquitin molecules can be cleaved from ubiquitin-modified proteins by a superfamily of DUBs (Fig. 1a) [10].

By regulating the quantity and quality of modified proteins, ubiquitination mediates almost all vital biological processes in eukaryotic cells, such as embryonic development, cell cycle control, and immunity [11]. If the ubiquitination process is abnormal, the disruption of protein homeostasis can ultimately lead to cellular dysfunction or even carcinogenesis [12, 13]. Notably, studies indicate that ubiquitination is widely involved in the regulation of the TME [14, 15]. Given the complexity and significance of the TME and ubiquitination, insights into the exact mechanisms by which ubiquitination regulates the TME would not only shed light on tumorigenesis but also help identify potential therapeutic targets. The present review first summarizes how E3 ligases and DUBs dynamically regulate a variety of key cellular proteins that play critical roles in the TME. Then, the latest and attractive techniques that take full advantage of the inherent ubiquitin‒proteasome system (UPS) to degrade targeted oncoproteins and reeducate the TME are introduced.

### E3 ubiquitin ligases and degrons within substrate proteins

There are over 600 identified E3 ligases, and these proteins are the largest and most critical components of the three classes of enzymes, determining the specificity and efficiency of substrate ubiquitination [16]. Increasing evidence suggests that abnormalities in E3 activity and subcellular localization are closely associated with various human diseases [12, 17]. Based on differences in the catalytic domains and process of ubiquitin transferring, E3 ligases are generally divided into three broad categories: RING (really interesting new gene), HECT (homologous to E6-AP carboxyl terminus), and RBR (ring-between-ring) E3 ligases (Fig. 1b) [18]. As the largest subgroup, RING E3 ligases can recruit Ub-loaded E2 ligases and substrates together to mediate the direct transfer of ubiquitin molecules [19]. In contrast, HECT E3 ligases possess a specific HECT domain containing a catalytic cysteine residue, which catalyzes the transesterification reaction of ubiquitin charged by E2 to form a transient Ub-E3 intermediate before transferring ubiquitin to the substrate [20]. Recently, hybrid RING/HECT E3 ligases known as RBR E3 ligases were found to simultaneously exhibit the functional characteristics of both RING and HECT types. Typically, RBR E3 ligases consist of two RING domains, in which the N-terminal RING1 domain can recruit Ub-loaded E2 in a RING E3 ligase-dependent manner, and the C-terminal RING2 domain possesses a catalytic cysteine similar to HECT E3 ligases [21].

Substrates are selectively recognized by E3 ligases in a key-lock manner. Therefore, what confers the specificity of the substrate? Degrons are a group of short peptide motifs that serve as the minimal but sufficient elements to allow recognition by the degradation mechanism within substrate proteins [22]. E3 ligases typically target the degrons of damaged, misfolded, mislocalized or redundant proteins for proteolysis via the UPS, which maintains protein homeostasis in cells [22, 23]. Intriguingly, fusing a degron element with a long-lived protein significantly reduces its half-life and confers instability, suggesting the potential transferability of degrons [24]. Degrons tend to be concentrated at both terminals of proteins rather than in the middle regions due to their intrinsically disordered property [25]. Although the first characterized degrons were the N-degrons at the N-terminals of short-lived proteins, an increasing number of C-terminal degrons have been identified [26–28]. Posttranscriptional modifications of degron, which are most commonly kinase-mediated phosphorylation, significantly alter the affinity of E3-degron binding, which can be exploited to integrate upstream signals and time protein degradation [29–31].

### The ubiquitin code and fate of proteins

Ubiquitin labeling of proteins is a sophisticated process known as the ubiquitin code that involves initial substrate lysine selection and ubiquitin chain elongation (Fig. 1c). Sometimes, one or more ubiquitin molecules are individually attached to substrate lysine residues in a one-to-one manner, which is defined as monoubiquitination or multi-monoubiquitination [32, 33]. As the simplest ubiquitination type, monoubiquitination was previously thought to regulate cellular processes, including DNA damage repair, histone functions, chromatin remodeling and receptor endocytosis, by affecting protein activity, localization and interaction [34, 35]. However, emerging evidence suggests an underestimated proteolytic role [36]. After monoubiquitination, another ubiquitin can use the different lysine residues or N-terminal methionine of linked ubiquitin as an anchor point, forming polyubiquitin chains. Because each ubiquitin molecule contains seven lysine residues and a particular N-terminal methionine (K6, K11, K27, K29, K33, K48, K63, and M1, respectively), insights into the diversity of ubiquitin linkages will help to further elucidate the relationship between ubiquitin encoding and functional consequences [37].

As the two most abundant linkage types, Lys48-linked polyubiquitination typically serves as a degradation signal for the 26S proteasome, while Lys63-linked chains play a nonproteolytic role in cellular processes [38]. Lys11-linked polyubiquitination participates in degradation of cell-cycle proteins during mitosis [39]. Similar to Lys63, Lys6-linked polyubiquitination is related to the DNA damage repair process but is not involved in degradation [40]. In addition to polyubiquitination, recent studies have identified the widespread presence of branched polyubiquitination, that is, the formation of branching chains on the basis of polyubiquitination. K11/K48-linked branched polyubiquitination is catalyzed by the APC/C E3 ligase complex, thereby promoting protein degradation to regulate the mitotic process [41, 42]. Notably, polyubiquitination chains exhibit different conformational flexibilities: extended or compact conformations, which potentially affect the fate of substrate proteins [43, 44].

### SUMOylation, neddylation and the deubiquitination process

Since the first discovery of ubiquitin in the 1970s, a series of ubiquitin-like (UBL) homologous small molecules have also been discovered, most notably SUMO and NEDD8. Each of the UBL modifiers can be catalyzed by an enzymatic cascade reaction similar to ubiquitination to covalently bind to substrate proteins [45]. Alternatively, ubiquitin-like proteins are able to mix in the polyubiquitin chain, which further increases the structural complexity of polyubiquitin chains [6]. In the human proteome, the SUMO family includes five SUMO paralogs (SUMO1-5), among which SUMO1, 2 and 3 exhibit ubiquitin-like protein modification functions [46]. Although E3 SUMO ligases are far fewer in number than E3 ubiquitin ligases, there are more than 500 SUMO substrates [47]. By regulating the stability, activity, and localization of these downstream substrates, SUMOylation orchestrates various vital facets of cellular processes [46]. Analogously, NEDD8-mediated posttranscriptional modification is known as neddylation, and cullin-family members are the best-known neddylation substrates. As the key subunit of Cullin-RING ligases (CRLs), the neddylation of cullin proteins can stabilize and activate the ubiquitination activity of CRLs [48, 49].

Similar to other posttranscriptional modifications, such as phosphorylation and acetylation, cellular protein ubiquitination appears to be a dynamic and reversible process that involves antagonism between E3 ligases and DUBs to precisely regulate intracellular protein homeostasis [50]. As a specific kind of protease, DUBs are capable of hydrolyzing the iso-peptide bond on polyubiquitin chains or ubiquitylated substrates and the particular peptide bond between ubiquitin and the N-terminal Met of another ubiquitin [51]. According to their sequence and domain homology, more than 100 DUBs in the human genome can be categorized into seven major families: USP, UCH, OTU, MJD, ZUFSP, MINDY, and JAMM [52]. In addition to reversing or reediting ubiquitin signals, DUBs play a vital role in processing ubiquitin precursors and cleaving polyubiquitin chains to maintain an adequate free ubiquitin pool [53]. Coincidentally, some cysteine proteases can remove UBL molecules from substrates; sentrin/SUMO-specific proteases (SENPs) can selectively target SUMO conjugation in humans [54].

### Major components of the tumor microenvironment

**Fig. 2:**
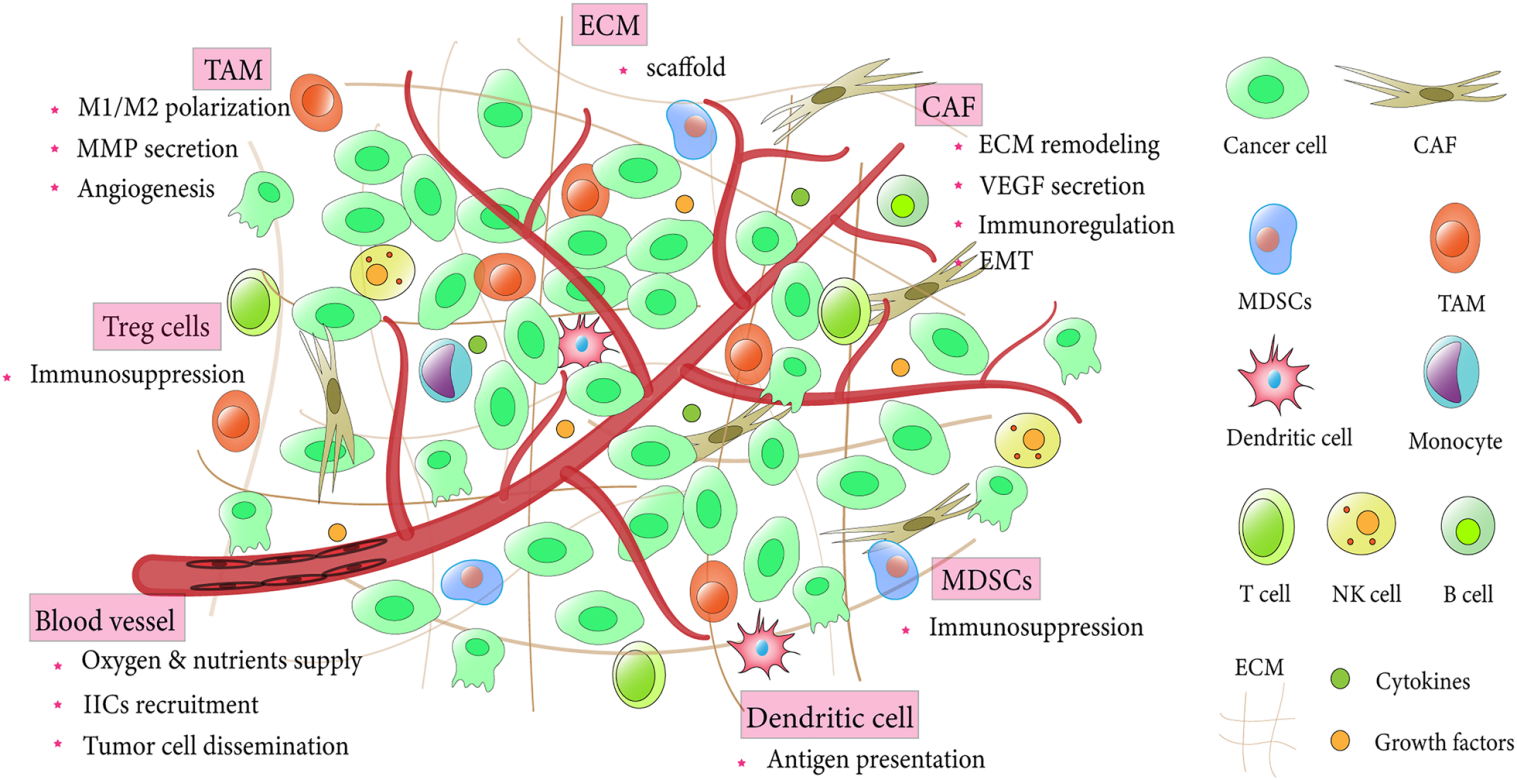
Major components of the tumor microenvironment. The tumor microenvironment refers to a complex and tumorigenic ecosystem comprised of multiple cell types (immune cells, fibroblasts, endothelial cells), as well as extracellular components including extracellular matrix (ECM) and secreted mediators (growth factors and cytokines, chemokines). Tumor cells can communicate bidirectionally with the surrounding tumor microenvironment and are nourished by limited or poorly differentiated blood vessels

Although cancer-related studies have previously focused on alterations in downstream proto-oncogenic pathways due to aberrant genetic mutations in cancer cells, the contribution of the TME to tumor phenotypes has received increasing attention in the last two decades. In addition to malignant tumor cells, the TME is composed of multiple stromal cells and complex noncellular constituents, which are nourished by a limited or poorly differentiated vasculature [2]. Stromal cells mainly include infiltrating immune cells (IICs), cancer-associated fibroblasts (CAFs), and cancer-associated endothelial cells (CAEs); ECM and other secreted mediators such as growth factors and cytokines are the most representative acellular components (Fig. 2) [1]. In the TME, bidirectional communication between tumor cells and their surrounding stroma collectively reconstructs the ectopic and tumorigenic organoid niches that not only participate in primary tumor growth and survival but also facilitate tumor progression, metastasis and drug resistance [3, 55].

#### Cancer-associated endothelial cells

In 1971, Judah Folkman first proposed a revolutionary viewpoint that neoplasm growth depended on angiogenesis [56]. Angiogenesis is involved in the activation of cancer‑associated endothelial cells from the preexisting vasculature to form new blood vessels to meet the cellular demands for oxygen and nutrients under physiological and pathological conditions. As the tumor expands (in diameter), the incompatibility between vascular supply and tumor metabolism leads to hypoxia in the TME, which in turn induces the synthesis of a series of proangiogenic factors in tumor cells and the surrounding stroma [57]. Among these proangiogenic factors, vascular endothelial growth factor-A (VEGF-A), a potent proangiogenic factor, directly initiates proangiogenic signaling pathways in vascular endothelial cells by binding to its receptor VEGFR2 (VEGF receptor 2) [58, 59]. However, excessive and sustained VEGF-VEGFR2 signaling in malignances leads to architecturally abnormal angiogenesis, which is characterized by irregular shape, poor differentiation, loose interendothelial contact, a defective basement membrane and reduced pericyte coverage [60]. These features are not only detrimental to adequate vascular perfusion of the tumor area but also greatly impair the vascular barrier, which allows blood extravasation and metastatic dissemination of cancer cells under high interstitial fluid pressure.

#### CAFs

As a major stromal cell type, CAFs can remodel the TME through the synthesis and degradation of its components, release diverse growth factors and cytokines to drive angiogenesis and tumor progression, and interfere with therapeutic responses by secreting soluble mediators [61]. Histopathological studies have indicated an association between CAF abundance and adverse clinical outcomes among a variety of human malignances [62, 63]. CAFs are initially derived from local tissue fibroblasts that are activated by diverse tumor-derived stimuli and can be further amplified via cell proliferation, resulting in the dominant source of CAFs [64]. In addition, the differentiation of mesenchymal stem cells and the conversion from resident epithelial or endothelial cells via epithelial-mesenchymal transition (EMT) or endothelial-mesenchymal transition (EndMT) has also been observed [65, 66]. Recently, the role of CAFs in immunoregulation has been gradually appreciated. By secreting various cytokines, such as CXCL12, and expressing specific surface molecules, CAFs participate in the recruitment, accumulation and maturation of Treg cells, thus inhibiting T effector responses [67]. Despite the potential immune-promoting effects of CAFs, their superior immunosuppressive effect has been widely accepted.

#### Immune cells

As early as 1863, Rudolf Virchow observed leukocyte infiltration in neoplastic tissues and further speculated the inflammatory origin of cancer [68]. Since then, a myriad of studies have been dedicated to elucidating the association between chronic inflammation and tumors, which are referred to as “wounds that do not heal”. The chronic inflammatory response suggests that the immune system does not ignore the tumor as a heterogeneous new organism but attempts to restore tissue homeostasis instead by damaging tumor cells [69]. These inflammatory environments contain a wide spectrum of IICs, which can be roughly divided into innate and adaptive immune cells. Among these IICs, tumor-associated macrophages (TAMs) are the most abundant innate immune cells, originating from bone marrow-derived monocytes and resident macrophages [70]. Currently, the subtype classification of TAMs proposed by Mills’ group is widely accepted, in which TAMs are categorized into M1 (classically activated) and M2 (alternatively activated) subtypes according to different polarization states [71]. M1 macrophages are induced by Th1 cytokines, particularly IFN-γ, and are involved in Th1-type immune responses, as they can mediate antigen presentation and secrete nitric oxide, reactive oxygen species (ROS), interleukins, and TNF-α, which are proinflammatory and antitumorigenic. In contrast, M2 macrophages, which are activated by Th2 cytokines such as IL-4, IL-10 and TGF-β, participate in Th2-type immune responses and show widespread anti-inflammatory and protumorigenic effects [72–74].

A critical hallmark of cancer is that immune cells in the TME become defective in response to tumor cells, and the host’s immune system becomes ineffective. Regulatory T (Treg) cells called suppressive T cells are a small class of specific CD4 + T cells with high CD25 expression, constituting 5–10% of human peripheral CD4 + T cells [75, 76]. Under physiological conditions, Treg cells are responsible for inhibiting the activation of self-reactive lymphocytes and maintaining tissue homeostasis via peripheral immune tolerance. However, Treg cell infiltration into the TME is thought to prevent tumor-associated antigen presentation and suppress the antitumor immune response [77]. In 2003, three laboratories simultaneously identified Forkhead Box P3 (Foxp3) as a core transcription factor in Treg cells, which dominates Treg cell development and immunosuppressive function [78–80]. Noticeably, deletions or mutations in Foxp3 genes cause vigorous peripheral infiltration of T effector cells in mice and severe autoimmune disease in humans [80, 81]. Similar to Treg cells, myeloid-derived suppressor cells (MDSCs) also compromise major mechanisms of immunosurveillance to promote tumor progression; these cells are a heterogeneous population of precursors of macrophages, dendritic cells, and granulocytes [82].

### Role of ubiquitination and SUMOylation in TME modulation

**Fig. 3:**
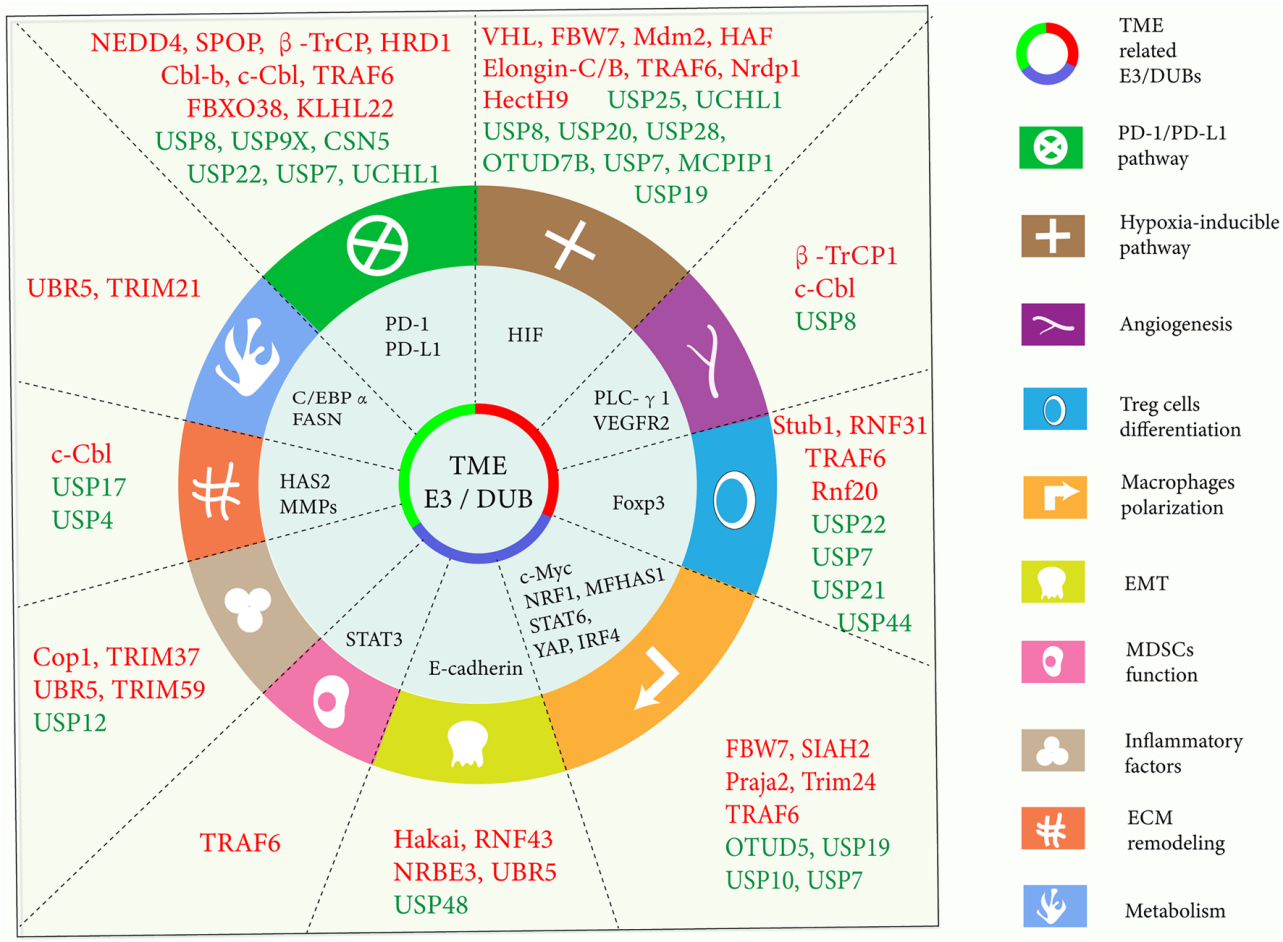
The role of E3 ligases and DUBs in the TME. The UPS deeply regulates the immunosuppressive TME, involving a variety of biological processes such as immune escape, angiogenesis, hypoxia, EMT, and degeneration of immune cells and stromal cells. Mechanistically, E3 ligases (shown in red) and DUBs (shown in blue) collectively target core factors, enzymes and transcription factors in these pro-tumorigenic processes

Abundant evidence has indicated that the tumorigenesis and progression are related to a variety of protumorigenic processes in the TME, including immune escape, angiogenesis, hypoxia, and the degeneration of immune cells and stromal cells. The complex interaction between the TME and tumor cells contributes to these biological changes in multiple components of the TME. Although the mechanism of the malignant transformation of the TME is complex, it often requires a series of core intracellular and extracellular factors, enzymes and transcription factors mediating signaling pathways to control the function of TME. Thus, identifying these key proteins and understanding the factors that affect their protein levels can provide ideas for studying the mechanism of TME formation and provide clues for subsequent drug discovery. Ubiquitin ligase-induced ubiquitination, which is the most important pathway that regulates protein degradation in cells, cooperates with DUBs to maintain protein homeostasis. Many studies have shown that the UPS closely regulates these biological processes and maintains the TME in an immunosuppressive state (Fig. 3). The E3 ubiquitin ligases and DUBs that regulate these key proteins, especially in specific TME, are summarized (Table 1).

**Table 1:** Summary of E3 ligases and deubiquitinases involved in the tumor microenvironment

Abbreviation	Full name	Substrate	Refs
E3 ligases			
NEDD4	Neuronal precursor cell expressed developmentally downregulated 4	PD-L1	[92]
SPOP	Speckle-type BTB/POZ protein	PD-L1	[30]
β-TrCP	β-transducin repeat-containing protein	PD-L1, VEGFR2	[94, 135]
HRD1	HMG-coA reductase degradation protein 1	PD-L1	[95]
Cbl-b	Casitas B lymphoma-b		[96]
c-Cbl	c-Caritas B cell lymphoma	PD-1, PLC-γ1, MMP2	[96, 103, 139, 203]
TRAF6	Tumor necrosis factor receptor-associated factor 6	PD-L1, HIF-1α, STAT6, Foxp3, STAT3	[97, 118, 149, 160, 188]
FBXO38	F-box protein 38	PD-1	[104, 105]
KLHL22	Kelch-like family member 22	PD-1	[106]
VHL	Von Hippel-Lindau	HIF-1α	[110]
Siah1/2	Seven in absentia homologue 1/2	PHDs	[111]
FBW7	F-box and WD repeat domain-containing 7	HIF-1α, c-Myc	[112, 145]
Mdm2	Mouse double minute 2	HIF-1α	[114, 115]
HAF	Hypoxia-associated factor	HIF-1α	[116]
Nrdp1	Neuregulin receptor degradation protein-1	USP8	[123]
HectH9		USP7	[125]
β-TrCP1	β-transducin repeat-containing protein 1	VEGFR2	[134]
SIAH2	Seven in absentia homolog 2	NRF1	[146]
Praja2		MFHAS1	[147]
Trim24	Tripartite motif containing protein 24	CBP	[148]
Stub1	STIP1 homology and U-box-containing protein 1	Foxp3	[157]
RNF31	Ring finger protein 31	Foxp3	[158]
Rnf20	Ring finger protein 20	Foxp3	[161]
Hakai		E-cadherin	[177]
RNF43	RING finger protein 43	E-cadherin	[179]
NRBE3	Novel RB E3 ubiquitin ligase	RB	[180]
UBR5	ubiquitin protein ligase E3 component n-recognin 5	Snail1, Twist, FBP1	[182, 204]
TRIM21	Tripartite motif containing protein 21	FASN	[206]
Deubiquitinases			
USP8	Ubiquitin-specific protease 8	PD-L1, HIF-1α, VEGFR2	[97, 121, 136]
USP9X	Ubiquitin-specific peptidase 9, X-linked	PD-L1	[98]
CSN5	COP9 signalosome 5	PD-L1	[99]
USP22	Ubiquitin-specific protease 22	PD-L1, Foxp3	[100, 161]
USP7	Ubiquitin-specific protease 7	PD-L1, HIF-1α, Foxp3, Tip60	[101, 125, 162, 163]
USP28	Ubiquitin-specific protease 28	HIF-1α	[113]
USP25	Ubiquitin-specific protease 25	HIF-1α	[119]
UCHL1	Ubiquitin C-terminal hydrolase L1	HIF-1α	[120]
USP20	Ubiquitin-specific protease 20	HIF-1α	[122]
MCPIP1	Monocyte chemoattractant protein-induced protein 1	HIF-1α	[126]
USP19	Ubiquitin-specific protease 19	NLRP3	[127, 152]
OTUD5	ovarian tumor deubiquitinase 5	YAP	[151]
USP10	Ubiquitin-specific protease 10	NLRP7	[153]
USP44	Ubiquitin-specific protease 44	Foxp3	[164]
USP21	Ubiquitin-specific protease 21	Foxp3	[165]
USP48	Ubiquitin-specific protease 48	TRAF2	[181]
USP17	Ubiquitin-specific protease 17	HAS2	[199]
USP4	Ubiquitin-specific protease 4	HAS2	[199]

#### PD-1/PD-L1 pathway

Immune responses mediated by T-lymphocytes are indispensable means against tumor cells, involving the formation of CD8 + cytotoxic T cells and the acquisition of helper functions by CD4 + cells [2]. Unfortunately, certain tumors have evolved various escape mechanisms to evade immune surveillance, one of which involves the activation of inhibitory receptors known as immune checkpoints on the surface of these T-effector cells [83, 84]. The binding of related ligands to immune checkpoints can negatively regulate T-cell migration, proliferation and antitumor activity, which is characterized by an exhausted phenotype [85]. In the TME, the PD-1/PD-L1 axis is an important representative immune checkpoint pathway; its potential clinical value has attracted considerable attention in recent years [86, 87]. PD-L1 (programmed death‑ligand 1) is widely expressed on the surface of cancer cells, ICCs including myeloid cells and T cells, and even tumor-associated nerves, and its expression is higher than that in adjacent normal tissues [88, 89].

Increasing evidence has revealed that multiple E3 ligases play critical roles in regulating PD-1/PD-L1 levels via ubiquitination [90, 91]. In bladder cancer cells, NEDD4, a HECT-type E3 ligase, can be phosphorylated by fibroblast growth factor receptor 3 to increase its Lys48-linked polyubiquitination activity for PD-L1 [92]. PD-L1 also serves as a substrate for the adaptor protein SPOP in Cullin3-Ring E3 ligases. Intriguingly, PD-L1 abundance is dynamically regulated during cell cycle progression because the Cyclin D-CDK4-mediated phosphorylation of SPOP effectively protects SPOP from degradation by APC/CCdh1 E3 ligase [30]. Skp1-Cul1-F-box (SCF) E3 ligase complexes, a well-characterized class of CRLs, need to target typical phospho-degrons in substrate proteins for ubiquitination. As the receptor subunits of SCF complexes, variable F-box proteins are able to selectively bind a distinct subset of substrates [93]. Phosphorylation mediated by glycogen synthase kinase 3β (GSK3β) is required to induce the recognition of PD-L1 by β-TrCP, a classic F-box protein. The glycosylation of PD-L1 can suppress contact between PD-L1 and GSK3β, indirectly stabilizing the PD-L1 protein [94]. In particular, PD-L1 in the endoplasmic reticulum (ER) can be phosphorylated at its S195 residue in a metformin-induced manner, which obstructs ER-to-Golgi translocation of PD-L1. Subsequently, aberrant ER accumulation enhances polyubiquitination-dependent proteasomal degradation of PD-L1 via the ER-associated degradation (ERAD) pathway, in which HRD1 plays an important role as an ERAD E3 ligase [95]. These four ubiquitin ligases all control PD-L1 protein levels by directly affecting the proteolysis of ubiquitin-modified PD-L1. However, Cbl-b and c-Cbl, which are monomeric RING-type E3 ligases, indirectly downregulate PD-L1 expression by inhibiting the activation of STAT3/AKT/ERK signaling in lung cancer [96]. In contrast, TRAF6 ligase positively regulates PD-L1 stability by catalyzing K63-linked polyubiquitination rather than the degradable K48-type linkage, which is abrogated by USP8 [97].

It is worth noting how DUBs reversely regulate PD-L1 polyubiquitination. Among them, USP9X, CSN5, USP22 and USP7 are responsible for the deubiquitination and stabilization of PD-L1 in tumor cells [98–101]. As a mechanism by which TNF-α inhibits T-cell surveillance, the TNF-α-activated transcription factor p65 mediates the transcriptional activation of CSN5, thereby inhibiting the ubiquitination and proteolysis of PD-L1 [99]. In particular, the UCH subgroup's DUB UCHL1 promotes PD-L1 transcription through the AKT-p65 axis, in which p65 recognizes and activates the promoter of the PD-L1 gene [102]. Analogously, various E3 ligases, including c-Cbl, FBXO38 and KLHL22, are involved in the K48-link ubiquitination of PD-1 (programmed death protein‑1). In tumor-infiltrating T cells, these E3 ligases are markedly reduced and T-cell function is significantly suppressed [103–106].

#### Hypoxia-inducible factor 1 (HIF-1)

In response to characteristic hypoxic stress in the TME, tumor cells trigger a variety of signaling pathways in which oxygen-sensitive HIF-1, a crucial transcriptional activator, can upregulate the transcription of over 100 hypoxia-inducible genes. These genes include VEGF, erythropoietin, lactate dehydrogenase A and glucose transporters, which orchestrate angiogenesis, erythropoiesis, anaerobic metabolism, tumor immune evasion, and other events in the tumor region [107–109]. HIF-1 functions as a heterodimer composed of a regulatory HIF-1α subunit and a constitutive HIF-1β subunit. Under normoxic conditions, HIF-1α is readily hydroxylated on its conserved Pro402/Pro564 residues and is subsequently ubiquitinated by VHL E3 ligase for proteolysis [110]. However, prolyl-hydroxylation of HIF-1α is significantly inhibited during hypoxia, partly due to the degradation of HIF-1α-targeting prolyl-hydroxylase proteins (PHDs) by the RING E3 ligases Siah1/2 [111].

Given the hypoxic state of tumors, it would be valuable to gain insight into how E3 ligases ubiquitinate HIF-1α through oxygen-independent mechanisms. Under hypoxic conditions, phosphorylated HIF-1α is catalyzed by GSK3β and can be ubiquitinated by FBW7 E3 ligase and degraded, and this effect is antagonized by USP28 to increase the half-life of HIF-1α [112, 113]. The E3 ligase Mdm2 is also enhanced by p52 to ubiquitinate HIF-1α during hypoxia [114]. Noticeably, Mdm2-mediated ubiquitination may occur only in the cytoplasm; activation of PI3K/AKT signaling induces the translocation of Mdm2 into the nucleus to interfere with ubiquitination [115]. HAF E3 ligase can control the basal levels of HIF-1α via ubiquitination independent of cellular oxygen tension [116]. In addition, receptor for activated protein C kinase 1 (RACK1) serves as an anchoring protein for HIF-1α to specifically recruit Elongin-C/B complex to HIF-1α, which that can be inhibited due to competitive binding between HSP90 and RACK1 [117]. Different from these degradation outcomes, RING E3 ligase TRAF6 can form a K63-linked polyubiquitin chain in an oxygen-independent condition, which contributes to the stability of HIF-1α [118].

As the only E3 ligase that mediates ubiquitination of HIF-1α in an oxygen-dependent manner, the VHL complex appears to be antagonized by several DUBs, including USP25 [119], UCHL1 [120], USP8 [121], and USP20 [122]. However, in the extremely hypoxic tumor microenvironment of pancreatic ductal adenocarcinoma, USP25 still deubiquitinate HIF-1α to abrogate its degradation [119]. Given that the mechanism of USP25 antagonism under this hypoxic condition was not described, whether there are other E3 ligases targeting HIF-1α that can be antagonized by USP25 in pancreatic ductal carcinoma deserves further investigation. In addition, Nrdp1 E3 ligase targets USP8 for degradation and can indirectly inhibit the deubiquitination of HIF-1α, thus destabilizing HIF-1α in ischemic neurons [123]. Surprisingly, Lys11-specific DUB OTUD7B stabilizes HIF-1α in the presence of VHL through chaperone-mediated autophagy rather than hydroxylation-dependent proteolysis [124]. In addition, the DUBs USP7, MCPIP1, and USP19 have been shown to deubiquitinate HIF-1α and increase its stability [125–127]. Among these factors, HectH9 E3 ligase in the HECT family can catalyze the K63-linked polyubiquitination of USP7 to increase its deubiquitinating functions, especially under the condition of intracellular hypoxia [125], and USP19 alleviates HIF-1α degradation independently of its catalytic activity [127]. Finally, SUMOylation of HIF-1α could replace prolyl-hydroxylation to promote VHL-mediated ubiquitination and proteolysis under hypoxia, and this effect could be reversed by SENP1 to regulate EPO production [128].

#### VEGF-A/VEGFR2 pathway

The activation of VEGFR2, a powerful receptor tyrosine kinase, is the most critical mechanism by which VEGF mediates and promotes tumor angiogenesis [129, 130]. After binding to VEGF-A, VEGFR2 triggers its own dimerization and catalyzes the autophosphorylation of multiple tyrosine residues in its intracellular kinase region, which is essential for the induction of pivotal downstream effectors such as phospholipase C-γ1 (PLC-γ1) [131]. Subsequently, activated VEGFR2 is rapidly removed from the cell surface and internalized by clathrin-mediated endocytosis [132]. Interestingly, it has been suggested that the ubiquitination of autophosphorylated VEGFR2 is involved in regulating its internalization and proteolysis [133]. Meyer et al. found that VEGFR2 contained an unstructured PEST sequence rich in serine and threonine residues, which is a potential recognition site for β-TrCP1 E3 ligases. In response to VEGF, the PEST sequence is phosphorylated at Ser1188/Ser1191, which is required for β-TrCP1-induced degradable polyubiquitination [134]. In another study, β-TrCP1 was shown to specifically recognize the C-terminal phosphodegrons of VEGFR2 in a casein kinase δ-dependent manner [135].

The balance between ubiquitination and deubiquitination is responsible for the intracellular trafficking, signal conduction and proteolysis of VEGFR2. The deubiquitinating enzyme USP8 can cleave the K48- and K63-type polyubiquitin chains of VEGFR2 to influence these processes [136]. USP8 depletion can significantly increase the retention of VEGFR2 in early endosomes and reduce its recycling to the plasma membrane. As a result, ubiquitinated VEGFR2 in early endosomes is subject to proteolysis, which impairs the activation of downstream Akt and ERK1/2 effectors, but not p38 MAPK or PLCγ1 [136]. In addition, SUMOylation was shown to preserve VEGFR2 localization in the Golgi apparatus, which could be reversed by SENP1-mediated deSUMOylation. SENP1-deficient endothelial cells fail to express sufficient VEGFR2 on their cell surface, which is not conducive to VEGFR2-mediated angiogenesis [137].

Peculiarly, tyrosine phosphorylation of PLC-γ1 induced by VEGFR2 in endothelial cells is required for cell proliferation by activating the downstream PKC/ERK pathway. On the one hand, the autophosphorylation of Tyr1173 on activated VEGFR2 is a major docking site for PLC-γ1 recruitment [138]. On the other hand, the autophosphorylation of Tyr1052 and Tyr1057 on VEGFR2 can directly bind to c-Cbl and phosphorylate its tyrosine to enhance ubiquitin-ligase activity [139]. In the tertiary complex, full activation of c-Cbl distinctly enhances the ubiquitination level of PLC-γ1 in a proteolysis-independent manner, which prevents tyrosine phosphorylation of PLC-γ1 [139, 140].

#### M1/M2-like macrophage polarization

M1- and M2-like macrophages in the TME exhibit two contradictory functional characteristics. However, macrophage polarization only indicates the activation state at a specific time [141]. When the stimulus in tissue microenvironment changes, the polarization state of macrophages will also be altered due to cellular plasticity [142, 143]. Mechanistically, a variety of key transcription factors can stimulate upstream signaling pathways to induce different macrophage phenotypes by regulating the transcriptional activity of different genes [144]. The UPS has been reported to target different transcription factors to regulate the polarization states of macrophages. For example, FBW7 is a tumor suppressor that inhibits macrophage transformation to the M2 type by degrading the oncoprotein c-Myc [145]. As a master transcription factor of mitochondria‑related genes, nuclear respiratory factor 1 (NRF1) can also be ubiquitinated by the hypoxia-activated E3 ligase SIAH2. Subsequently, the downregulation of nuclear-encoded mitochondrial gene expression due to NRF1 degradation contributes to M2-like polarization, which may be associated with metabolic remodeling [146].

In addition, E3 ligase-mediated ubiquitination regulates macrophage polarization in a nondegradable manner. Praja2 polyubiquitinates and stabilizes the oncoprotein MFHAS1, which is critical for inducing M1-like macrophage polarization by upregulating the TLR2/JNK/p38/NF-κB pathway [147]. Moreover, STAT6 has been recognized as a pivotal transcription factor that drives macrophage M2 polarization [144]. Acetyltransferase CREB-binding protein (CBP) can be ubiquitinated by Trim24 E3 ligase to promote its binding to STAT6, which is required for STAT6 acetylation. Acetylation of STAT6 dramatically inhibits its transcriptional activity towards M2 macrophage-specific genes [148]. During IL-4-induced activation of M2 macrophages, the E3 ligase TRAF6 promotes K63-linked ubiquitination of STAT6 and inhibits K48-linked ubiquitination of STAT6, ultimately avoiding the degradation of STAT6. Unexpectedly, K63 ubiquitination mediated by TRAF6 is dispensable for STAT6 stability, and the suppression of K48 ubiquitination is independent of its E3 ligase activity [149]. As another key transcription factor in IL-4-induced activation of M2 macrophages, KLF4 can be SUMOylated to increase its transcriptional activity [150].

In recent years, we have also recognized that multiple DUBs promote macrophage M2 polarization through different mechanisms. Among these DUBs, OTUD5 of the OTU subfamily deubiquitinates Yes-associated protein (YAP) to maintain its stability in macrophages. As a significant transcriptional coactivator in the Hippo pathway, YAP was first shown to be involved in M2 macrophage polarization, which further contributed to the invasive properties of triple-negative breast cancer (TNBC) [151]. As for the transcription factor interferon regulatory factor 4 (IRF4) in M2 macrophages, USP19 can competitively block the binding between IRF4 and p62 by deubiquitinating and stabilizing NLRP3, thus protecting IRF4 from p62-mediated selective autophagic degradation [152]. Furthermore, USP10-mediated stabilization of NLRP7 promotes TAM polarization through NF-κB pathway-induced CCL2 secretion [153]. By screening 51 DUB genes, USP7 was shown to be abundant in M2 but not M1 macrophages. Specific silencing of USP7 can effectively upregulate the M1-related p38 MAPK pathway, resulting in reeducating TAMs to the M1 type [154].

#### Foxp3 in Treg cells

The differentiation and immunosuppressive capabilities of Treg cells are characteristically programmed by a specific gene expression profile, which is predominantly governed by the transcription factor Foxp3. The phenotypes of Treg cells and effector T cells are unstable and can be switched according to intracellular Foxp3 abundance [78, 80]. Multiple posttranscriptional modification mechanisms regulate Foxp3 activity, stability and localization, among which ubiquitination plays an important role [155, 156]. To date, three E3 ubiquitin ligases have been identified that specifically ubiquitinate Foxp3: Stub1, RNF31, and TRAF6. As the first E3 ligase that was shown to mediate Foxp3 ubiquitination, Stub1 is upregulated and translocates from the cytoplasm to the nucleus in response to multiple cytokines and LPS-mediated inflammatory stimuli. In the Foxp3 complex, Hsp70 is an essential mediator that can recruit stress-activated Stub1 to target its partner Foxp3 for degradation [157]. RNF31, a component of the linear ubiquitin chain assembly complex, is responsible for catalyzing multi-monoubiquitination of Foxp3 at eight lysine residues [158]. This atypical ubiquitination modification contributes to the stability of Foxp3, which is indispensable for maintaining the immunosuppressive phenotype of tumor-infiltrating Treg cells [158, 159]. Similar to the positive regulation of Foxp3 by RNF31, TRAF6 can catalyze K63-type polyubiquitination at Lys262, thereby cancelling the perinuclear isolation of Foxp3 and promoting its translocation to nuclear transcription sites [160].

In a CRISPR-based gene knockout screen of approximately 500 nuclear factors, the deubiquitinase USP22 and E3 ligase Rnf20 were recognized as reciprocal ubiquitin switches that positively and negatively regulate Foxp3 expression in mouse primary Treg cells, respectively [161]. In addition, USP7, USP21 and USP44 can directly recognize Foxp3 and remove its K48-linked polyubiquitin chains to maintain Foxp3 homeostasis. When Foxp3 is deubiquitinated, USP7 can also deubiquitinate and stabilize the histone/protein acetyltransferase Tip60, which is required for the acetylation, dimerization and function of Foxp3 [162, 163]. Notably, TGF-β-induced USP44 exhibits an obvious synergistic effect with USP7, and the coexpression of USP44 and USP7 almost completely eliminates Foxp3 polyubiquitination [164]. A total of 7 lysine residues on Foxp3 are potential targets of USP21-mediated deubiquitination [165]. Furthermore, stable Foxp3 can recognize the promoter of the USP21 gene and upregulate its transcriptional activity, thus forming a positive feedback loop between Foxp3 and USP21 [166, 167]. Similar to the master transcription factor Foxp3, the basic leucine zipper transcription repressor BACH2 is required for maintaining the differentiation and immunosuppressive activity of Treg cells by repressing effector T-cell-specific transcriptional programs [168]. DeSUMOylation of BACH2 is mediated by the ROS-SENP3-BACH2 axis, facilitates the nuclear localization of BACH2 and maintains Treg cell-specific gene signatures [169].

#### EMT associated with cancer stem cells and CAFs

EMT is a reversible process characterized by epithelial cells acquiring mesenchymal properties. Phenotypically, epithelial cells undergoing EMT lose cell junctions and apical-basal polarity and reshape their cytoskeleton to gain a new shape and better motility [170]. During tumorigenesis, the formation of cancer stem cells and some CAFs has been confirmed to require EMT induction. Cancer stem cells, which are a rare population of tumor cells, have the potential for self-renewal and multilineage differentiation, which are closely related to the initiation and relapse of multiple human tumors [171–173]. The most significant molecular hallmark of EMT is the disappearance of the transmembrane protein E-cadherin that mediates epithelial cell adhesions and preserves epithelial integrity [174]. In response to cytokines such as TGF-β and multiple stress cues, EMT-related pathways can be hijacked to activate downstream EMT-inducing transcription factors (EMT-TFs) [175]. These EMT-TFs include the Snail family members Snail1 and Snail2 (also known as Snail and Slug), the Zeb family members Zeb1 and Zeb2, and Twist1, all of which have the ability to inhibit E-cadherin transcription [175, 176].

In epithelial cells, the ubiquitination of E-cadherin is catalyzed by the E3 ligase Hakai and can be endocytosed, which may eventually lead to its recycling back to the cell membrane or destruction [177, 178]. Before being ubiquitinated by Hakai, E-cadherin needs to be tyrosine phosphorylated by the tyrosine kinase c-Src [177]. Similarly, c-Src-mediated tyrosine phosphorylation is indispensable for RNF43-mediated ubiquitination of E-cadherin in lung adenocarcinoma [179]. As an E3 ligase targeting retinoblastoma protein (RB), NRBE3 negatively regulates E-cadherin transcription in breast cancer mainly through RB-dependent mechanisms [180]. The deubiquitinase USP48 is an indirect regulator of E-cadherin expression. In response to TNF-α, GSK3β-mediated serine phosphorylation can further enhance the deubiquitination activity of USP48, which in turn stabilizes the second messenger adaptor TRAF2 in the TNF-α pathway and ultimately suppresses E-cadherin expression [181]. In addition, the E3 ubiquitin ligase UBR5 not only regulates the growth, metastasis and immune response of TNBC but also affects the expression of E-cadherin. Deletion of UBR5 reduces intracellular E-cadherin levels and the aberrant EMT phenotype. After excluding the effect of UBR5 on promoter hypermethylation of the E-cadherin gene, the degradation of EMT-TFs including Snail1 and Twist is thought to be a potential mechanism by which UBR5 positively regulates E-cadherin [182].

It is worth noting that the imbalance in the dynamic regulation in EMT-TFs by ubiquitin modification is an important factor that induces EMT. In epithelial cells, most EMT-TFs, which are short-lived proteins, are susceptible to rapid UPS-mediated degradation and thus remain at very low levels [183]. Numerous F-box proteins have been shown to participate in EMT by targeting EMT-TFs degradation [184]. Broader E3 ligases and DUBs have also been comprehensively described in several reviews [185–187]. Therefore, further elucidation of the specific mechanisms of the UPS-DUBs system in EMT-TFs degradation and how it fails in tumorigenesis can help identify attractive targets for inhibiting tumor metastasis.

#### Mediator production, ECM and cancer metabolism

In MDSCs, the E3 ligase TRAF6 catalyzes K63-linked polyubiquitination of the core transcription factor STAT3, which is a prerequisite for the phosphorylation and activation of STAT3. With the substantially increased expression of TRAF6 in tumor-infiltrating MDSCs, abnormally activated STAT3 signaling impairs the maturation of myeloid progenitor cells and promotes the proliferation and immunosuppressive activity of MDSCs [188]. Moreover, the specific STAT3 phosphatase CD45 in MDSCs can be SUMOylated to attenuate its phosphatase activity and maintain the phosphorylation and activation of STAT3, which can be reversed by SENP1 [189]. In addition, chronic inflammation in the TME can activate multiple inflammatory pathways in tumor cells, tumor stromal cells, and ICCs, thereby promoting the release of inflammatory mediators, including growth factors, cytokines, chemokines, and proangiogenic factors [190, 191]. The ubiquitin ligases Cop1, TRIM37, and UBR5 have been shown to modulate the production of key chemokines and cytokines in tumor cells to promote the recruitment and activation of immunosuppressive macrophages [192–194]. Downregulation of the deubiquitinase USP12 in lung cancer cells promotes the hyperactivation of NF-κB, a positive regulator of inflammation, and generates a tumor-promoting secretome by reducing the stability of PPM1B [195]. In particular, the ubiquitin ligase TRIM59 can be transported from tumor cells to macrophages via exosomes to increase the secretion of IL-1β by macrophages [196].

As an acellular component of the TME, ECM produced by stromal cells possesses an intricate fiber network that plays a key role in tumor growth, invasion and metastasis [197]. The activity of hyaluronan synthase 2 (HAS2), which is responsible for hyaluronan synthesis, in CAFs is associated with monoubiquitination at Lys190 [198]. The ubiquitination of HAS2 was also shown to be removed by diverse DUBs, among which USP17 and USP4 effectively remove polyubiquitin chains and monoubiquitin molecules from HAS2, respectively [199]. In solid tumors, the ECM often becomes highly dysregulated, resulting in the loss of normal matrix organization and homeostasis [200]. As a major class of ECM-remodeling proteinases, matrix metalloproteinases (MMPs) can extensively degrade matrix proteins and are closely related to the malignant migration of tumor cells and the selective release of bound signaling mediators [201]. With the continuous in-depth research on the regulatory mechanism of MMPs, a variety of E3 ubiquitin ligases have been shown to target MMPs [202]. For example, c-Cbl facilitates tumor invasion and metastasis by upregulating MMP2 expression in human glioma [203].

Finally, UPS-mediated regulation of tumor metabolism can respond to metabolic stress in the TME and meet the growth and survival needs of cancer cells. In response to characteristic nutrient deprivation and hypoxia in the TME, pancreatic cancer cells fully utilize aerobic glycolysis for energy in an UBR5-dependent manner. Mechanistically, upregulated UBR5 ubiquitinates the transcription factor C/EBPα of fructose-1,6-bisphosphatase (FBP1) for proteolysis and reduces the levels of the rate-limiting enzyme FBP1 in gluconeogenesis to promote aerobic glycolysis [204]. Hexokinase 2, which is the first rate-limiting enzyme of glycolysis, is expressed in aggressive tumors and can bind to the outer membrane of mitochondria to promote glycolysis and reduce mitochondrial respiration [205]. The affinity of hexokinase 2 to mitochondria can be regulated by SUMOylation, and de-SUMOylated hexokinase 2, which is catalyzed by SENP1, preferably binds to mitochondria [205]. TRIM21 can ubiquitinate and degrade acetylated fatty acid synthase (FASN). FASN is a rate-limiting enzyme complex that catalyzes de novo lipogenesis, and its level is frequently increased in human hepatocellular carcinoma, which is accompanied by diminished FASN acetylation and is conducive to tumor cell growth [206].

### Potential strategies for reeducating the TME through the UPS

**Fig. 4:**
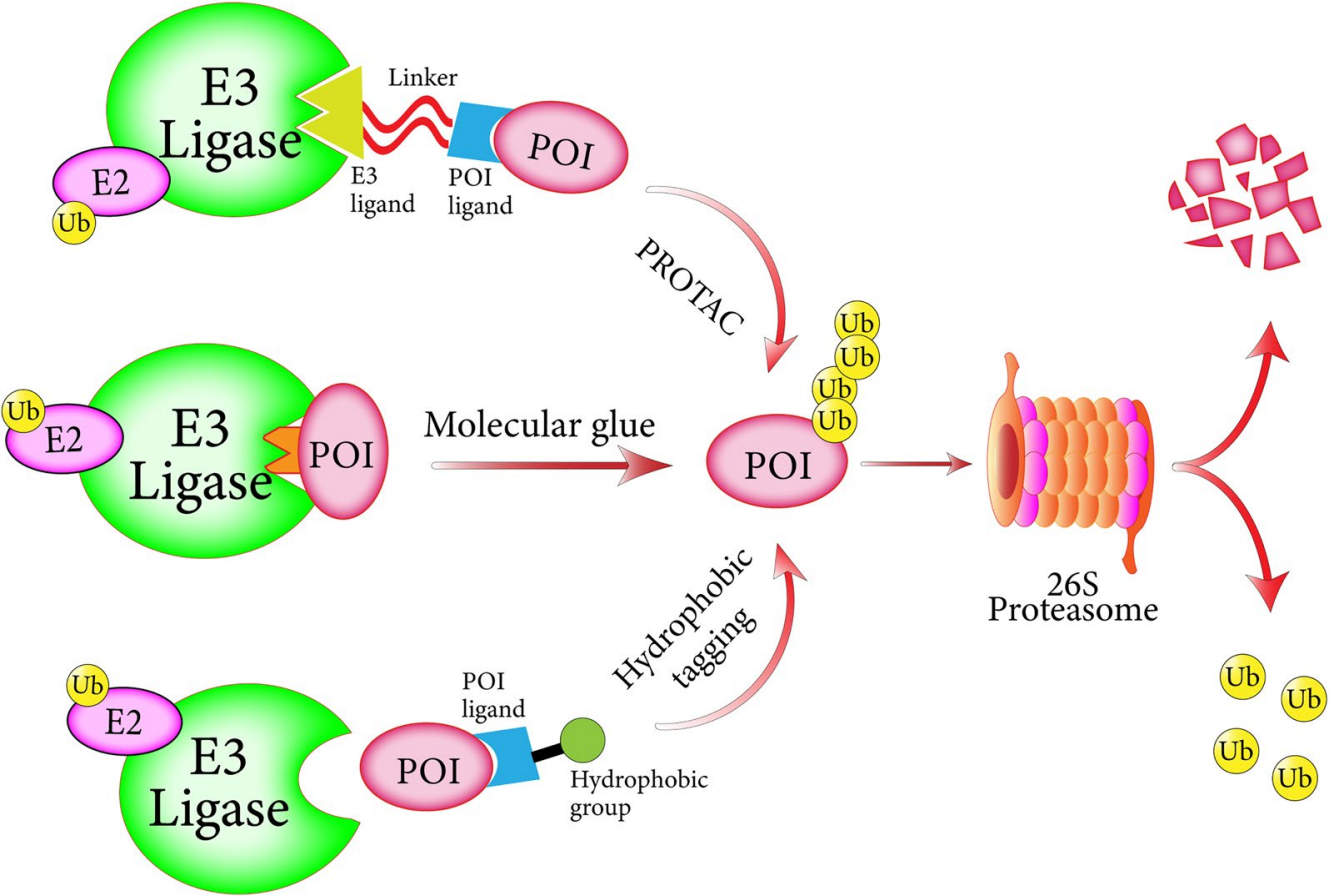
Small-molecule degraders hijacking the inherent UPS. PROTACs, molecular glues and hydrophobic tags all hijack intracellular E3 ligases and recruit targeted proteins for ubiquitination and subsequent degradation. Both PROTACs and hydrophobic tags are heterobifunctional small-molecule degraders containing a ligand of a protein of interest, which can recruit E3 ligase through specific ligand and hydrophobic group, respectively. Molecular glues, a class of proximity-inducing small molecules, can modify the substrate-recognizing site of E3 ligase by direct contact, allowing it interaction with neosubstrates

Increasing evidence has shown that reforming the TME into an anticancer environment is a promising therapeutic strategy, given the plasticity of the multiple stromal cells and its importance in cancer progression and drug resistance [1, 207–210]. Most drugs currently targeting the TME, such as immunotherapies and antiangiogenic drugs, are not very effective and typically only benefit one subset of patients [211–213]. In addition, a variety of oncoproteins, especially transcription factors lacking enzymatic activity, have been regarded as undruggable targets according to traditional occupancy-driven pharmacology. As accumulating evidence points to the critical role of ubiquitination in the TME, targeting key mediators with the full use of the inherent UPS seems to be an attractive strategy for anticancer drug development. Notably, extensive examination of small-molecule degraders has made it possible to hijack intracellular E3 ubiquitin-ligase machinery for targeted protein degradation (Fig. 4) [214, 215].

Since the innovative concept was first proposed in 2001, proteolysis targeting chimeras (PROTACs) have received considerable attention as a powerful tool with the potential to revolutionize pharmaceutical research [216, 217]. Typically, PROTACs are heterobifunctional small-molecule degraders whose two ligands, which are bridged by a short linker, can simultaneously recruit a specific E3 ligase and a POI (protein of interest). PROTAC technology, which relies on the formation of the E3-PROTAC-POI ternary complex, can theoretically achieve ubiquitination and subsequent proteolysis of any protein target by merely changing the POI ligand. Due to their stable structure, PROTACs can be recycled for continuous POI degradation [218]. In addition, a unique feature and advantage of PROTACs lies in its inherent selectivity of intracellular E3 ligases. The E3 ligases VHL, MDM2, CRBN, and IAP have been reported to be successfully recruited by PROTACs, and the potential of other E3 ligases as candidates needs to be further verified [219, 220]. Given that E3 ligase activity is related to expression levels in different tumor cell types and subcellular localization, the selection of appropriate E3 ligands based on different tumor types provides a feasible strategy for specific recognition of tumor tissues and maximum protection of other normal tissues in follow-up PROTACs design [215]. The latest advance in the PROTAC technology field involves the orally bioavailable PROTAC drugs ARV-110 and ARV-471, which have entered phase I clinical trials and target androgen and estrogen receptors, respectively; these drugs have shown encouraging efficacy, safety, and pharmacokinetic profiles in the treatment of prostate and breast cancer [221–223]. With the extensive research of PROTAC technology in cancer treatment, PROTACs present unique advantages over traditional inhibitors and antibodies by targeting TME pathways [224, 225].

Analogous to PROTACs, hydrophobic tags are heterobifunctional small-molecule degraders that link POI ligands to highly hydrophobic groups [226]. Different from ligand-mediated E3 ligase recruitment, hydrophobic tagging technology takes advantage of hydrophobic groups to mimic a misfolded state and induce the unfolded protein response. Under physiological conditions, the exposure of hydrophobic side-chains can activate the chaperone machinery that assists in the refolding of misfolded proteins [227]. However, the hydrophobic groups in hydrophobic tags cannot be repaired by chaperones, eventually leading to UPS-mediated POI clearance. Molecular glues, which are a class of proximity-inducing small molecules, can modify the substrate-recognizing site of an E3 ligase through direct contact, allowing for interactions with neosubstrates. Thalidomide and its derivatives, which are known as immunomodulatory drugs, have been widely researched as molecular glues through which substrate-receptor CRBN can recognize as many as 13 neosubstrates, which plays a critical role in the treatment of multiple myeloma [228–231]. It should be pointed out that extracellular proteins in the TME cannot be disregarded due to their levels and importance, but the targeted degradation methods introduced above seem inadequate. To address this challenge, Bertozzi's team and Spiegel's team proposed Lysosomal-Targeting Chimeras (LYTACs) and Molecular Degraders of Extracellular proteins through the Asialoglycoprotein receptor (MoDE-As) technology, respectively [232, 233]. Mechanistically, LYTACs and MoDE-As drive the internalization and subsequent lysosomal degradation of extracellular proteins through recycling receptors.

## Conclusions and perspectives

In the present review, we mainly summarized the proteolytic ubiquitination signals in the tumor microenvironment and tumorigenesis. Ubiquitin signaling is involved in most steps in the TME, and its dysregulation eventually leads to tumorigenesis and tumor progression. Thus, targeting the ubiquitin system has provided good therapeutic strategies for cancer treatments and have achieved some FDA-approved POIs, including small molecule PROTACs. PROTAC has several advantages, such as good cell permeability, solubility, and easy for oral administration. Until now, only several E3 ligases have been used in PROTACs, and more promising candidate E3 ligases are being explored to expand the way of cancer treatment. However, the specific role of ubiquitination in immune cells, such as T cells and macrophages, is still unclear, especially regarding immunotherapies targeting ubiquitination. On the other hand, we do not completely understand the structural analysis of target proteins and the dynamic process of tumorigenesis, and most of these POIs work well in cell culture or animal models but not well enough in clinical trials. Thus, in-depth understanding the functions of the UPS in the TME can expand and diversify the range of novel anticancer strategies, and more clinical studies should be conducted in the future.

## Data Availability

All data generated during this study are included in this article.

## Abbreviations

CAEs: Cancer-associated endothelial cells
CAFs: Cancer-associated fibroblasts
CRLs: Cullin-RING ligases
DUBs: Deubiquitinases
ECM: Extracellular matrix
EMT: Epithelial-mesenchymal transition
EMT-TFs: EMT-inducing transcription factors
EndMT: Endothelial-mesenchymal transition
ER: Endoplasmic reticulum
ERAD: ER-associated degradation
FASN: Fatty acid synthase
FBP1: Fructose-1,6-bisphosphatase
Foxp3: Forkhead box P3
GSK3β: Glycogen synthase kinase 3β
HAS2: Hyaluronan synthase 2
HECT: Homologous to E6-AP carboxyl terminus
HIF-1: Hypoxia-inducible factor 1
IICs: Infiltrating immune cells
IRF4: Interferon regulatory factor 4
LYTACs: Lysosomal-targeting chimeras
MDSCs: Myeloid-derived suppressor cells
MMPs: Matrix metalloproteinases
MoDE-As: Molecular degraders of extracellular proteins through the asialoglycoprotein receptor
NRF1: Nuclear respiratory factor 1
PD‑1: Programmed death protein‑1
PD‑L1: Programmed death‑ligand 1
PHDs: Prolyl-hydroxylase proteins
PLC-γ1: Phospholipase C-γ1
POI: Protein of interest
PROTACs: Proteolysis targeting chimeras
RACK1: Receptor of activated protein C kinase 1
RB: Retinoblastoma protein
RBR: Ring-between-ring
RING: Really interesting new gene
ROS: Reactive oxygen species
SCF: Skp1-Cul1-F-box
SENP: Sentrin/SUMO-specific proteases
TAMs: Tumor-associated macrophages
TME: Tumor microenvironment
TNBC: Triple-negative breast cancer
Treg cells: Regulatory T cells
Ub: Ubiquitin
UBL: Ubiquitin-like
UPS: Ubiquitin–proteasome system
VEGF-A: Vascular endothelial growth factor-A
VEGFR2: VEGF receptor 2
YAP: Yes-associated protein

## References

[1] Bejarano L, Jordāo MJC, Joyce JA (2021). Therapeutic targeting of the tumor microenvironment. Cancer Discov.

[2] Quail DF, Joyce JA (2013). Microenvironmental regulation of tumor progression and metastasis. Nat Med.

[3] Wu T, Dai Y (2017). Tumor microenvironment and therapeutic response. Cancer Lett.

[4] Jia Q, Wang A, Yuan Y, Zhu B, Long H (2022). Heterogeneity of the tumor immune microenvironment and its clinical relevance. Exp Hematol Oncol.

[5] Duan G, Walther D (2015). The roles of post-translational modifications in the context of protein interaction networks. PLoS Comput Biol.

[6] Swatek KN, Komander D (2016). Ubiquitin modifications. Cell Res.

[7] Hershko A, Ciechanover A (1998). The ubiquitin system. Annu Rev Biochem.

[8] Ye Y, Rape M (2009). Building ubiquitin chains: E2 enzymes at work. Nat Rev Mol Cell Biol.

[9] Chang SC, Zhang BX, Ding JL (2022). E2–E3 ubiquitin enzyme pairing - partnership in provoking or mitigating cancers. Biochim Biophys Acta Rev Cancer.

[10] Clague MJ, Urbé S, Komander D (2019). Breaking the chains: deubiquitylating enzyme specificity begets function. Nat Rev Mol Cell Biol.

[11] Rape M (2018). Ubiquitylation at the crossroads of development and disease. Nat Rev Mol Cell Biol.

[12] Popovic D, Vucic D, Dikic I (2014). Ubiquitination in disease pathogenesis and treatment. Nat Med.

[13] Huang N, Sun X, Li P, Liu X, Zhang X, Chen Q (2022). TRIM family contribute to tumorigenesis, cancer development, and drug resistance. Exp Hematol Oncol.

[14] Chang SC, Ding JL (2018). Ubiquitination and SUMOylation in the chronic inflammatory tumor microenvironment. Biochim Biophys Acta Rev Cancer.

[15] Zhou L, Jiang Y, Luo Q, Li L, Jia L (2019). Neddylation: a novel modulator of the tumor microenvironment. Mol Cancer.

[16] Buetow L, Huang DT (2016). Structural insights into the catalysis and regulation of E3 ubiquitin ligases. Nat Rev Mol Cell Biol.

[17] Li G, Ci W, Karmakar S, Chen K, Dhar R, Fan Z (2014). SPOP promotes tumorigenesis by acting as a key regulatory hub in kidney cancer. Cancer Cell.

[18] Morreale FE, Walden H (2016). Types of ubiquitin ligases. Cell.

[19] Deshaies RJ, Joazeiro CA (2009). RING domain E3 ubiquitin ligases. Annu Rev Biochem.

[20] Rotin D, Kumar S (2009). Physiological functions of the HECT family of ubiquitin ligases. Nat Rev Mol Cell Biol.

[21] Eisenhaber B, Chumak N, Eisenhaber F, Hauser MT (2007). The ring between ring fingers (RBR) protein family. Genome Biol.

[22] Zheng N, Shabek N (2017). Ubiquitin ligases: structure, function, and regulation. Annu Rev Biochem.

[23] Yeh CW, Huang WC, Hsu PH, Yeh KH, Wang LC, Hsu PW (2021). The C-degron pathway eliminates mislocalized proteins and products of deubiquitinating enzymes. Embo j.

[24] Natsume T, Kanemaki MT (2017). Conditional degrons for controlling protein expression at the protein level. Annu Rev Genet.

[25] Lobanov MY, Furletova EI, Bogatyreva NS, Roytberg MA, Galzitskaya OV (2010). Library of disordered patterns in 3D protein structures. PLoS Comput Biol.

[26] Bachmair A, Finley D, Varshavsky A (1986). In vivo half-life of a protein is a function of its amino-terminal residue. Science.

[27] Chen X, Liao S, Makaros Y, Guo Q, Zhu Z, Krizelman R (2021). Molecular basis for arginine C-terminal degron recognition by Cul2(FEM1) E3 ligase. Nat Chem Biol.

[28] Koren I, Timms RT, Kula T, Xu Q, Li MZ, Elledge SJ (2018). The eukaryotic proteome is shaped by E3 ubiquitin ligases targeting C-Terminal degrons. Cell.

[29] Liu P, Cong X, Liao S, Jia X, Wang X, Dai W (2022). Global identification of phospho-dependent SCF substrates reveals a FBXO22 phosphodegron and an ERK-FBXO22-BAG3 axis in tumorigenesis. Cell Death Differ.

[30] Zhang J, Bu X, Wang H, Zhu Y, Geng Y, Nihira NT (2018). Cyclin D-CDK4 kinase destabilizes PD-L1 via cullin 3-SPOP to control cancer immune surveillance. Nature.

[31] Chen Y, Shao X, Cao J, Zhu H, Yang B, He Q (2021). Phosphorylation regulates cullin-based ubiquitination in tumorigenesis. Acta Pharm Sin B.

[32] Fuseya Y, Fujita H, Kim M, Ohtake F, Nishide A, Sasaki K (2020). The HOIL-1L ligase modulates immune signalling and cell death via monoubiquitination of LUBAC. Nat Cell Biol.

[33] Aisenberg WH, McCray BA, Sullivan JM, Diehl E, DeVine LR, Alevy J (2022). Multiubiquitination of TRPV4 reduces channel activity independent of surface localization. J Biol Chem.

[34] Gupta-Rossi N, Six E, LeBail O, Logeat F, Chastagner P, Olry A (2004). Monoubiquitination and endocytosis direct gamma-secretase cleavage of activated Notch receptor. J Cell Biol.

[35] Ulrich HD, Walden H (2010). Ubiquitin signalling in DNA replication and repair. Nat Rev Mol Cell Biol.

[36] Braten O, Livneh I, Ziv T, Admon A, Kehat I, Caspi LH (2016). Numerous proteins with unique characteristics are degraded by the 26S proteasome following monoubiquitination. Proc Natl Acad Sci USA.

[37] Suryadinata R, Roesley SN, Yang G, Sarčević B (2014). Mechanisms of generating polyubiquitin chains of different topology. Cells.

[38] Grice GL, Nathan JA (2016). The recognition of ubiquitinated proteins by the proteasome. Cell Mol Life Sci.

[39] Matsumoto ML, Wickliffe KE, Dong KC, Yu C, Bosanac I, Bustos D (2010). K11-linked polyubiquitination in cell cycle control revealed by a K11 linkage-specific antibody. Mol Cell.

[40] Morris JR, Solomon E (2004). BRCA1: BARD1 induces the formation of conjugated ubiquitin structures, dependent on K6 of ubiquitin, in cells during DNA replication and repair. Hum Mol Genet.

[41] Meyer HJ, Rape M (2014). Enhanced protein degradation by branched ubiquitin chains. Cell.

[42] Oh E, Mark KG, Mocciaro A, Watson ER, Prabu JR, Cha DD (2020). Gene expression and cell identity controlled by anaphase-promoting complex. Nature.

[43] Komander D, Reyes-Turcu F, Licchesi JD, Odenwaelder P, Wilkinson KD, Barford D (2009). Molecular discrimination of structurally equivalent Lys 63-linked and linear polyubiquitin chains. EMBO Rep.

[44] Satoh T, Sakata E, Yamamoto S, Yamaguchi Y, Sumiyoshi A, Wakatsuki S (2010). Crystal structure of cyclic Lys48-linked tetraubiquitin. Biochem Biophys Res Commun.

[45] Cappadocia L, Lima CD (2018). Ubiquitin-like protein conjugation: structures, chemistry, and mechanism. Chem Rev.

[46] Sahin U, de Thé H, Lallemand-Breitenbach V (2022). Sumoylation in physiology, pathology and therapy. Cells.

[47] Lamoliatte F, Caron D, Durette C, Mahrouche L, Maroui MA, Caron-Lizotte O (2014). Large-scale analysis of lysine SUMOylation by SUMO remnant immunoaffinity profiling. Nat Commun.

[48] Schwechheimer C (2018). NEDD8-its role in the regulation of Cullin-RING ligases. Curr Opin Plant Biol.

[49] Zheng YC, Guo YJ, Wang B, Wang C, Mamun MAA, Gao Y (2021). Targeting neddylation E2s: a novel therapeutic strategy in cancer. J Hematol Oncol.

[50] Harrigan JA, Jacq X, Martin NM, Jackson SP (2018). Deubiquitylating enzymes and drug discovery: emerging opportunities. Nat Rev Drug Discov.

[51] Komander D, Clague MJ, Urbé S (2009). Breaking the chains: structure and function of the deubiquitinases. Nat Rev Mol Cell Biol.

[52] Snyder NA, Silva GM (2021). Deubiquitinating enzymes (DUBs): regulation, homeostasis, and oxidative stress response. J Biol Chem.

[53] Cheng J, Guo J, North BJ, Wang B, Cui CP, Li H (2019). Functional analysis of deubiquitylating enzymes in tumorigenesis and development. Biochim Biophys Acta Rev Cancer.

[54] Kunz K, Piller T, Müller S (2018). SUMO-specific proteases and isopeptidases of the SENP family at a glance. J Cell Sci.

[55] Hanahan D, Coussens LM (2012). Accessories to the crime: functions of cells recruited to the tumor microenvironment. Cancer Cell.

[56] Folkman J (1971). Tumor angiogenesis: therapeutic implications. N Engl J Med.

[57] LaGory EL, Giaccia AJ (2016). The ever-expanding role of HIF in tumour and stromal biology. Nat Cell Biol.

[58] Apte RS, Chen DS, Ferrara N (2019). VEGF in signaling and disease: beyond discovery and development. Cell.

[59] Claesson-Welsh L, Welsh M (2013). VEGFA and tumour angiogenesis. J Intern Med.

[60] Bergers G, Benjamin LE (2003). Tumorigenesis and the angiogenic switch. Nat Rev Cancer.

[61] Park D, Sahai E, Rullan A (2020). SnapShot: cancer-associated fibroblasts. Cell.

[62] Heichler C, Scheibe K, Schmied A, Geppert CI, Schmid B, Wirtz S (2020). STAT3 activation through IL-6/IL-11 in cancer-associated fibroblasts promotes colorectal tumour development and correlates with poor prognosis. Gut.

[63] Bremnes RM, Dønnem T, Al-Saad S, Al-Shibli K, Andersen S, Sirera R (2011). The role of tumor stroma in cancer progression and prognosis: emphasis on carcinoma-associated fibroblasts and non-small cell lung cancer. J Thorac Oncol.

[64] Sahai E, Astsaturov I, Cukierman E, DeNardo DG, Egeblad M, Evans RM (2020). A framework for advancing our understanding of cancer-associated fibroblasts. Nat Rev Cancer.

[65] Zeisberg EM, Potenta S, Xie L, Zeisberg M, Kalluri R (2007). Discovery of endothelial to mesenchymal transition as a source for carcinoma-associated fibroblasts. Cancer Res.

[66] Kalluri R, Neilson EG (2003). Epithelial-mesenchymal transition and its implications for fibrosis. J Clin Invest.

[67] Costa A, Kieffer Y, Scholer-Dahirel A, Pelon F, Bourachot B, Cardon M (2018). Fibroblast heterogeneity and immunosuppressive environment in human breast cancer. Cancer Cell.

[68] Balkwill F, Mantovani A (2001). Inflammation and cancer: back to Virchow?. Lancet.

[69] Dvorak HF (2015). Tumors: wounds that do not heal-redux. Cancer Immunol Res.

[70] Cassetta L, Pollard JW (2020). Tumor-associated macrophages. Curr Biol.

[71] Mills CD, Kincaid K, Alt JM, Heilman MJ, Hill AM (2000). M-1/M-2 macrophages and the Th1/Th2 paradigm. J Immunol.

[72] Qian BZ, Pollard JW (2010). Macrophage diversity enhances tumor progression and metastasis. Cell.

[73] Wu K, Lin K, Li X, Yuan X, Xu P, Ni P (2020). Redefining tumor-associated macrophage subpopulations and functions in the tumor microenvironment. Front Immunol.

[74] Wang L, He T, Liu J, Tai J, Wang B, Chen Z (2021). Pan-cancer analysis reveals tumor-associated macrophage communication in the tumor microenvironment. Exp Hematol Oncol.

[75] Gershon RK, Kondo K (1970). Cell interactions in the induction of tolerance: the role of thymic lymphocytes. Immunology.

[76] Sakaguchi S, Sakaguchi N, Asano M, Itoh M, Toda M (1995). Immunologic self-tolerance maintained by activated T cells expressing IL-2 receptor alpha-chains (CD25) breakdown of a single mechanism of self-tolerance causes various autoimmune diseases. J Immunol.

[77] Zou W (2006). Regulatory T cells, tumour immunity and immunotherapy. Nat Rev Immunol.

[78] Hori S, Nomura T, Sakaguchi S (2003). Control of regulatory T cell development by the transcription factor Foxp3. Science.

[79] Fontenot JD, Gavin MA, Rudensky AY (2003). Foxp3 programs the development and function of CD4+CD25+ regulatory T cells. Nat Immunol.

[80] Khattri R, Cox T, Yasayko SA, Ramsdell F (2003). An essential role for Scurfin in CD4+CD25+ T regulatory cells. Nat Immunol.

[81] Wildin RS, Ramsdell F, Peake J, Faravelli F, Casanova JL, Buist N (2001). X-linked neonatal diabetes mellitus, enteropathy and endocrinopathy syndrome is the human equivalent of mouse scurfy. Nat Genet.

[82] Li K, Shi H, Zhang B, Ou X, Ma Q, Chen Y (2021). Myeloid-derived suppressor cells as immunosuppressive regulators and therapeutic targets in cancer. Signal Transduct Target Ther.

[83] Jhunjhunwala S, Hammer C, Delamarre L (2021). Antigen presentation in cancer: insights into tumour immunogenicity and immune evasion. Nat Rev Cancer.

[84] Qin S, Xu L, Yi M, Yu S, Wu K, Luo S (2019). Novel immune checkpoint targets: moving beyond PD-1 and CTLA-4. Mol Cancer.

[85] Burugu S, Dancsok AR, Nielsen TO (2018). Emerging targets in cancer immunotherapy. Semin Cancer Biol.

[86] Yi M, Niu M, Zhang J, Li S, Zhu S, Yan Y (2021). Combine and conquer: manganese synergizing anti-TGF-β/PD-L1 bispecific antibody YM101 to overcome immunotherapy resistance in non-inflamed cancers. J Hematol Oncol.

[87] Yi M, Zheng X, Niu M, Zhu S, Ge H, Wu K (2022). Combination strategies with PD-1/PD-L1 blockade: current advances and future directions. Mol Cancer.

[88] Herbst RS, Soria JC, Kowanetz M, Fine GD, Hamid O, Gordon MS (2014). Predictive correlates of response to the anti-PD-L1 antibody MPDL3280A in cancer patients. Nature.

[89] Mo RJ, Han ZD, Liang YK, Ye JH, Wu SL, Lin SX (2019). Expression of PD-L1 in tumor-associated nerves correlates with reduced CD8(+) tumor-associated lymphocytes and poor prognosis in prostate cancer. Int J Cancer.

[90] Hu X, Wang J, Chu M, Liu Y, Wang ZW, Zhu X (2021). Emerging role of ubiquitination in the regulation of PD-1/PD-L1 in cancer immunotherapy. Mol Ther.

[91] Yi M, Niu M, Xu L, Luo S, Wu K (2021). Regulation of PD-L1 expression in the tumor microenvironment. J Hematol Oncol.

[92] Jing W, Wang G, Cui Z, Xiong G, Jiang X, Li Y (2022). FGFR3 destabilizes PD-L1 via NEDD4 to control T-cell-mediated bladder cancer immune surveillance. Cancer Res.

[93] Wang Z, Liu P, Inuzuka H, Wei W (2014). Roles of F-box proteins in cancer. Nat Rev Cancer.

[94] Li CW, Lim SO, Xia W, Lee HH, Chan LC, Kuo CW (2016). Glycosylation and stabilization of programmed death ligand-1 suppresses T-cell activity. Nat Commun.

[95] Cha JH, Yang WH, Xia W, Wei Y, Chan LC, Lim SO (2018). Metformin Promotes Antitumor Immunity via Endoplasmic-Reticulum-Associated Degradation of PD-L1. Mol Cell.

[96] Wang S, Xu L, Che X, Li C, Xu L, Hou K (2018). E3 ubiquitin ligases Cbl-b and c-Cbl downregulate PD-L1 in EGFR wild-type non-small cell lung cancer. FEBS Lett.

[97] Xiong W, Gao X, Zhang T, Jiang B, Hu MM, Bu X (2022). USP8 inhibition reshapes an inflamed tumor microenvironment that potentiates the immunotherapy. Nat Commun.

[98] Jingjing W, Wenzheng G, Donghua W, Guangyu H, Aiping Z, Wenjuan W (2018). Deubiquitination and stabilization of programmed cell death ligand 1 by ubiquitin-specific peptidase 9, X-linked in oral squamous cell carcinoma. Cancer Med.

[99] Lim SO, Li CW, Xia W, Cha JH, Chan LC, Wu Y (2016). Deubiquitination and stabilization of PD-L1 by CSN5. Cancer Cell.

[100] Huang X, Zhang Q, Lou Y, Wang J, Zhao X, Wang L (2019). USP22 Deubiquitinates CD274 to suppress anticancer immunity. Cancer Immunol Res.

[101] Wang Z, Kang W, Li O, Qi F, Wang J, You Y (2021). Abrogation of USP7 is an alternative strategy to downregulate PD-L1 and sensitize gastric cancer cells to T cells killing. Acta Pharm Sin B.

[102] Mao R, Tan X, Xiao Y, Wang X, Wei Z, Wang J (2020). Ubiquitin C-terminal hydrolase L1 promotes expression of programmed cell death-ligand 1 in non-small-cell lung cancer cells. Cancer Sci.

[103] Lyle C, Richards S, Yasuda K, Napoleon MA, Walker J, Arinze N (2019). c-Cbl targets PD-1 in immune cells for proteasomal degradation and modulates colorectal tumor growth. Sci Rep.

[104] Meng X, Liu X, Guo X, Jiang S, Chen T, Hu Z (2018). FBXO38 mediates PD-1 ubiquitination and regulates anti-tumour immunity of T cells. Nature.

[105] Serman TM, Gack MU (2019). FBXO38 drives PD-1 to destruction. Trends Immunol.

[106] Zhou XA, Zhou J, Zhao L, Yu G, Zhan J, Shi C (2020). KLHL22 maintains PD-1 homeostasis and prevents excessive T cell suppression. Proc Natl Acad Sci U S A.

[107] Choudhry H, Harris AL (2018). Advances in hypoxia-inducible factor biology. Cell Metab.

[108] McGettrick AF, O'Neill LAJ (2020). The role of HIF in immunity and inflammation. Cell Metab.

[109] Tao J, Yang G, Zhou W, Qiu J, Chen G, Luo W (2021). Targeting hypoxic tumor microenvironment in pancreatic cancer. J Hematol Oncol.

[110] Levy AP, Levy NS, Iliopoulos O, Jiang C, Kaplin WG, Goldberg MA (1997). Regulation of vascular endothelial growth factor by hypoxia and its modulation by the von Hippel-Lindau tumor suppressor gene. Kidney Int.

[111] Nakayama K, Ronai Z (2004). Siah: new players in the cellular response to hypoxia. Cell Cycle.

[112] Cassavaugh JM, Hale SA, Wellman TL, Howe AK, Wong C, Lounsbury KM (2011). Negative regulation of HIF-1α by an FBW7-mediated degradation pathway during hypoxia. J Cell Biochem.

[113] Flügel D, Görlach A, Kietzmann T (2012). GSK-3β regulates cell growth, migration, and angiogenesis via Fbw7 and USP28-dependent degradation of HIF-1α. Blood.

[114] Ravi R, Mookerjee B, Bhujwalla ZM, Sutter CH, Artemov D, Zeng Q (2000). Regulation of tumor angiogenesis by p53-induced degradation of hypoxia-inducible factor 1alpha. Genes Dev.

[115] Joshi S, Singh AR, Durden DL (2014). MDM2 regulates hypoxic hypoxia-inducible factor 1α stability in an E3 ligase, proteasome, and PTEN-phosphatidylinositol 3-kinase-AKT-dependent manner. J Biol Chem.

[116] Koh MY, Darnay BG, Powis G (2008). Hypoxia-associated factor, a novel E3-ubiquitin ligase, binds and ubiquitinates hypoxia-inducible factor 1alpha, leading to its oxygen-independent degradation. Mol Cell Biol.

[117] Liu YV, Semenza GL (2007). RACK1 vs. HSP90: competition for HIF-1 alpha degradation vs. stabilization. Cell Cycle.

[118] Sun H, Li XB, Meng Y, Fan L, Li M, Fang J (2013). TRAF6 upregulates expression of HIF-1α and promotes tumor angiogenesis. Cancer Res.

[119] Nelson JK, Thin MZ, Evan T, Howell S, Wu M, Almeida B (2022). USP25 promotes pathological HIF-1-driven metabolic reprogramming and is a potential therapeutic target in pancreatic cancer. Nat Commun.

[120] Goto Y, Zeng L, Yeom CJ, Zhu Y, Morinibu A, Shinomiya K (2015). UCHL1 provides diagnostic and antimetastatic strategies due to its deubiquitinating effect on HIF-1α. Nat Commun.

[121] Troilo A, Alexander I, Muehl S, Jaramillo D, Knobeloch KP, Krek W (2014). HIF1α deubiquitination by USP8 is essential for ciliogenesis in normoxia. EMBO Rep.

[122] Li Z, Wang D, Messing EM, Wu G (2005). VHL protein-interacting deubiquitinating enzyme 2 deubiquitinates and stabilizes HIF-1alpha. EMBO Rep.

[123] Zhang Y, Yang K, Wang T, Li W, Jin X, Liu W (2017). Nrdp1 increases ischemia induced primary rat cerebral cortical neurons and pheochromocytoma cells apoptosis via downregulation of hif-1α protein. Front Cell Neurosci.

[124] Bremm A, Moniz S, Mader J, Rocha S, Komander D (2014). Cezanne (OTUD7B) regulates HIF-1α homeostasis in a proteasome-independent manner. EMBO Rep.

[125] Wu HT, Kuo YC, Hung JJ, Huang CH, Chen WY, Chou TY (2016). K63-polyubiquitinated HAUSP deubiquitinates HIF-1α and dictates H3K56 acetylation promoting hypoxia-induced tumour progression. Nat Commun.

[126] Sun P, Lu YX, Cheng D, Zhang K, Zheng J, Liu Y (2018). Monocyte chemoattractant protein-induced protein 1 targets hypoxia-inducible factor 1α to protect against hepatic ischemia/reperfusion injury. Hepatology.

[127] Altun M, Zhao B, Velasco K, Liu H, Hassink G, Paschke J (2012). Ubiquitin-specific protease 19 (USP19) regulates hypoxia-inducible factor 1α (HIF-1α) during hypoxia. J Biol Chem.

[128] Cheng J, Kang X, Zhang S, Yeh ET (2007). SUMO-specific protease 1 is essential for stabilization of HIF1alpha during hypoxia. Cell.

[129] Jain RK (2003). Molecular regulation of vessel maturation. Nat Med.

[130] Qin S, Li A, Yi M, Yu S, Zhang M, Wu K (2019). Recent advances on anti-angiogenesis receptor tyrosine kinase inhibitors in cancer therapy. J Hematol Oncol.

[131] Koch S, Claesson-Welsh L (2012). Signal transduction by vascular endothelial growth factor receptors. Cold Spring Harb Perspect Med.

[132] Lampugnani MG, Orsenigo F, Gagliani MC, Tacchetti C, Dejana E (2006). Vascular endothelial cadherin controls VEGFR-2 internalization and signaling from intracellular compartments. J Cell Biol.

[133] Bruns AF, Herbert SP, Odell AF, Jopling HM, Hooper NM, Zachary IC (2010). Ligand-stimulated VEGFR2 signaling is regulated by co-ordinated trafficking and proteolysis. Traffic.

[134] Meyer RD, Srinivasan S, Singh AJ, Mahoney JE, Gharahassanlou KR, Rahimi N (2011). PEST motif serine and tyrosine phosphorylation controls vascular endothelial growth factor receptor 2 stability and downregulation. Mol Cell Biol.

[135] Shaik S, Nucera C, Inuzuka H, Gao D, Garnaas M, Frechette G (2012). SCF(β-TRCP) suppresses angiogenesis and thyroid cancer cell migration by promoting ubiquitination and destruction of VEGF receptor 2. J Exp Med.

[136] Smith GA, Fearnley GW, Abdul-Zani I, Wheatcroft SB, Tomlinson DC, Harrison MA (2016). VEGFR2 trafficking, signaling and proteolysis is regulated by the ubiquitin isopeptidase USP8. Traffic.

[137] Zhou HJ, Xu Z, Wang Z, Zhang H, Zhuang ZW, Simons M (2018). SUMOylation of VEGFR2 regulates its intracellular trafficking and pathological angiogenesis. Nat Commun.

[138] Meyer RD, Latz C, Rahimi N (2003). Recruitment and activation of phospholipase Cgamma1 by vascular endothelial growth factor receptor-2 are required for tubulogenesis and differentiation of endothelial cells. J Biol Chem.

[139] Singh AJ, Meyer RD, Navruzbekov G, Shelke R, Duan L, Band H (2007). A critical role for the E3-ligase activity of c-Cbl in VEGFR-2-mediated PLCgamma1 activation and angiogenesis. Proc Natl Acad Sci USA.

[140] Rahimi N (2009). A role for protein ubiquitination in VEGFR-2 signalling and angiogenesis. Biochem Soc Trans.

[141] Zhu S, Yi M, Wu Y, Dong B, Wu K (2021). Roles of tumor-associated macrophages in tumor progression: implications on therapeutic strategies. Exp Hematol Oncol.

[142] Locati M, Curtale G, Mantovani A (2020). Diversity, mechanisms, and significance of macrophage plasticity. Annu Rev Pathol.

[143] Boutilier AJ, Elsawa SF (2021). Macrophage polarization states in the tumor microenvironment. Int J Mol Sci.

[144] Lawrence T, Natoli G (2011). Transcriptional regulation of macrophage polarization: enabling diversity with identity. Nat Rev Immunol.

[145] Zhong L, Zhang Y, Li M, Song Y, Liu D, Yang X (2020). E3 ligase FBXW7 restricts M2-like tumor-associated macrophage polarization by targeting c-Myc. Aging.

[146] Ma B, Cheng H, Mu C, Geng G, Zhao T, Luo Q (2019). The SIAH2-NRF1 axis spatially regulates tumor microenvironment remodeling for tumor progression. Nat Commun.

[147] Zhong J, Wang H, Chen W, Sun Z, Chen J, Xu Y (2017). Ubiquitylation of MFHAS1 by the ubiquitin ligase praja2 promotes M1 macrophage polarization by activating JNK and p38 pathways. Cell Death Dis.

[148] Yu T, Gan S, Zhu Q, Dai D, Li N, Wang H (2019). Modulation of M2 macrophage polarization by the crosstalk between Stat6 and Trim24. Nat Commun.

[149] Zhou C, Lu C, Pu H, Li D, Zhang L (2020). TRAF6 promotes IL-4-induced M2 macrophage activation by stabilizing STAT6. Mol Immunol.

[150] Wang K, Zhou W, Cai Q, Cheng J, Cai R, Xing R (2017). SUMOylation of KLF4 promotes IL-4 induced macrophage M2 polarization. Cell Cycle.

[151] Zhang Y, Fan Y, Jing X, Zhao L, Liu T, Wang L (2021). OTUD5-mediated deubiquitination of YAP in macrophage promotes M2 phenotype polarization and favors triple-negative breast cancer progression. Cancer Lett.

[152] Liu T, Wang L, Liang P, Wang X, Liu Y, Cai J (2021). USP19 suppresses inflammation and promotes M2-like macrophage polarization by manipulating NLRP3 function via autophagy. Cell Mol Immunol.

[153] Li B, Qi ZP, He DL, Chen ZH, Liu JY, Wong MW (2021). NLRP7 deubiquitination by USP10 promotes tumor progression and tumor-associated macrophage polarization in colorectal cancer. J Exp Clin Cancer Res.

[154] Dai X, Lu L, Deng S, Meng J, Wan C, Huang J (2020). USP7 targeting modulates anti-tumor immune response by reprogramming tumor-associated macrophages in lung cancer. Theranostics.

[155] Dong Y, Yang C, Pan F (2021). Post-translational regulations of foxp3 in treg cells and their therapeutic applications. Front Immunol.

[156] Yan Y, Huang L, Liu Y, Yi M, Chu Q, Jiao D (2022). Metabolic profiles of regulatory T cells and their adaptations to the tumor microenvironment: implications for antitumor immunity. J Hematol Oncol.

[157] Chen Z, Barbi J, Bu S, Yang HY, Li Z, Gao Y (2013). The ubiquitin ligase Stub1 negatively modulates regulatory T cell suppressive activity by promoting degradation of the transcription factor Foxp3. Immunity.

[158] Zhu F, Yi G, Liu X, Zhu F, Zhao A, Wang A (2018). Ring finger protein 31-mediated atypical ubiquitination stabilizes forkhead box P3 and thereby stimulates regulatory T-cell function. J Biol Chem.

[159] Teh CE, Lalaoui N, Jain R, Policheni AN, Heinlein M, Alvarez-Diaz S (2016). Linear ubiquitin chain assembly complex coordinates late thymic T-cell differentiation and regulatory T-cell homeostasis. Nat Commun.

[160] Ni X, Kou W, Gu J, Wei P, Wu X, Peng H (2019). TRAF6 directs FOXP3 localization and facilitates regulatory T-cell function through K63-linked ubiquitination. Embo j.

[161] Cortez JT, Montauti E, Shifrut E, Gatchalian J, Zhang Y, Shaked O (2020). CRISPR screen in regulatory T cells reveals modulators of Foxp3. Nature.

[162] van Loosdregt J, Fleskens V, Fu J, Brenkman AB, Bekker CP, Pals CE (2013). Stabilization of the transcription factor Foxp3 by the deubiquitinase USP7 increases Treg-cell-suppressive capacity. Immunity.

[163] Wang L, Kumar S, Dahiya S, Wang F, Wu J, Newick K (2016). Ubiquitin-specific protease-7 inhibition impairs Tip60-dependent Foxp3+ T-regulatory cell function and promotes antitumor immunity. EBioMedicine.

[164] Yang J, Wei P, Barbi J, Huang Q, Yang E, Bai Y (2020). The deubiquitinase USP44 promotes Treg function during inflammation by preventing FOXP3 degradation. EMBO Rep.

[165] Li Y, Lu Y, Wang S, Han Z, Zhu F, Ni Y (2016). USP21 prevents the generation of T-helper-1-like Treg cells. Nat Commun.

[166] Zhang J, Chen C, Hou X, Gao Y, Lin F, Yang J (2013). Identification of the E3 deubiquitinase ubiquitin-specific peptidase 21 (USP21) as a positive regulator of the transcription factor GATA3. J Biol Chem.

[167] Pannu J, Belle JI, Förster M, Duerr CU, Shen S, Kane L (2015). Ubiquitin specific protease 21 is dispensable for normal development, hematopoiesis and lymphocyte differentiation. PLoS ONE.

[168] Roychoudhuri R, Hirahara K, Mousavi K, Clever D, Klebanoff CA, Bonelli M (2013). BACH2 represses effector programs to stabilize T(reg)-mediated immune homeostasis. Nature.

[169] Yu X, Lao Y, Teng XL, Li S, Zhou Y, Wang F (2018). SENP3 maintains the stability and function of regulatory T cells via BACH2 deSUMOylation. Nat Commun.

[170] Yang J, Antin P, Berx G, Blanpain C, Brabletz T, Bronner M (2020). Guidelines and definitions for research on epithelial-mesenchymal transition. Nat Rev Mol Cell Biol.

[171] Wang W, Liu W, Chen Q, Yuan Y, Wang P (2022). Targeting CSC-related transcription factors by E3 ubiquitin ligases for cancer therapy. Semin Cancer Biol.

[172] Dong B, Li S, Zhu S, Yi M, Luo S, Wu K (2021). MiRNA-mediated EMT and CSCs in cancer chemoresistance. Exp Hematol Oncol.

[173] Huang Y, Hong W, Wei X (2022). The molecular mechanisms and therapeutic strategies of EMT in tumor progression and metastasis. J Hematol Oncol.

[174] Bruner HC, Derksen PWB (2018). Loss of E-cadherin-dependent cell-cell adhesion and the development and progression of cancer. Cold Spring Harb Perspect Biol.

[175] Lamouille S, Xu J, Derynck R (2014). Molecular mechanisms of epithelial-mesenchymal transition. Nat Rev Mol Cell Biol.

[176] Wu K, Chen K, Wang C, Jiao X, Wang L, Zhou J (2014). Cell fate factor DACH1 represses YB-1-mediated oncogenic transcription and translation. Cancer Res.

[177] Fujita Y, Krause G, Scheffner M, Zechner D, Leddy HE, Behrens J (2002). Hakai, a c-Cbl-like protein, ubiquitinates and induces endocytosis of the E-cadherin complex. Nat Cell Biol.

[178] Pece S, Gutkind JS (2002). E-cadherin and Hakai: signalling, remodeling or destruction?. Nat Cell Biol.

[179] Zhang Y, Sun L, Gao X, Guo A, Diao Y, Zhao Y (2019). RNF43 ubiquitinates and degrades phosphorylated E-cadherin by c-Src to facilitate epithelial-mesenchymal transition in lung adenocarcinoma. BMC Cancer.

[180] Zheng T, Lu M, Wang T, Zhang C, Du X (2018). NRBE3 promotes metastasis of breast cancer by down-regulating E-cadherin expression. Biochim Biophys Acta Mol Cell Res.

[181] Li S, Wang D, Zhao J, Weathington NM, Shang D, Zhao Y (2018). The deubiquitinating enzyme USP48 stabilizes TRAF2 and reduces E-cadherin-mediated adherens junctions. Faseb j.

[182] Liao L, Song M, Li X, Tang L, Zhang T, Zhang L (2017). E3 Ubiquitin Ligase UBR5 Drives the Growth and Metastasis of Triple-Negative Breast Cancer. Cancer Res.

[183] Díaz VM, Viñas-Castells R, de GarcíaHerreros A (2014). Regulation of the protein stability of EMT transcription factors. Cell Adh Migr.

[184] Díaz VM, de Herreros AG (2016). F-box proteins: Keeping the epithelial-to-mesenchymal transition (EMT) in check. Semin Cancer Biol.

[185] Basu B, Ghosh MK (2022). Ubiquitination and deubiquitination in the regulation of epithelial-mesenchymal transition in cancer: shifting gears at the molecular level. Biochim Biophys Acta Mol Cell Res.

[186] Wang ZW, Hu X, Ye M, Lin M, Chu M, Shen X (2020). NEDD4 E3 ligase: functions and mechanism in human cancer. Semin Cancer Biol.

[187] Rodríguez-Alonso A, Casas-Pais A, Roca-Lema D, Graña B, Romay G, Figueroa A (2020). Regulation of epithelial-mesenchymal plasticity by the E3 ubiquitin-ligases in cancer. Cancers.

[188] Song G, Zhang Y, Tian J, Ma J, Yin K, Xu H (2021). TRAF6 regulates the immunosuppressive effects of myeloid-derived suppressor cells in tumor-bearing host. Front Immunol.

[189] Huang X, Zuo Y, Wang X, Wu X, Tan H, Fan Q (2019). SUMO-specific protease 1 is critical for myeloid-derived suppressor cell development and function. Cancer Res.

[190] Crusz SM, Balkwill FR (2015). Inflammation and cancer: advances and new agents. Nat Rev Clin Oncol.

[191] Mantovani A, Allavena P, Sica A, Balkwill F (2008). Cancer-related inflammation. Nature.

[192] Wang X, Tokheim C, Gu SS, Wang B, Tang Q, Li Y (2021). In vivo CRISPR screens identify the E3 ligase Cop1 as a modulator of macrophage infiltration and cancer immunotherapy target. Cell.

[193] Song M, Yeku OO, Rafiq S, Purdon T, Dong X, Zhu L (2020). Tumor derived UBR5 promotes ovarian cancer growth and metastasis through inducing immunosuppressive macrophages. Nat Commun.

[194] Do TT, Yeh CC, Wu GW, Hsu CC, Chang HC, Chen HC (2022). TRIM37 promotes pancreatic cancer progression through modulation of cell growth, migration, invasion, and tumor immune microenvironment. Int J Mol Sci.

[195] Yang Z, Xu G, Wang B, Liu Y, Zhang L, Jing T (2021). USP12 downregulation orchestrates a protumourigenic microenvironment and enhances lung tumour resistance to PD-1 blockade. Nat Commun.

[196] Liang M, Chen X, Wang L, Qin L, Wang H, Sun Z (2020). Cancer-derived exosomal TRIM59 regulates macrophage NLRP3 inflammasome activation to promote lung cancer progression. J Exp Clin Cancer Res.

[197] Mohan V, Das A, Sagi I (2020). Emerging roles of ECM remodeling processes in cancer. Semin Cancer Biol.

[198] Karousou E, Kamiryo M, Skandalis SS, Ruusala A, Asteriou T, Passi A (2010). The activity of hyaluronan synthase 2 is regulated by dimerization and ubiquitination. J Biol Chem.

[199] Mehić M, de Sa VK, Hebestreit S, Heldin CH, Heldin P (2017). The deubiquitinating enzymes USP4 and USP17 target hyaluronan synthase 2 and differentially affect its function. Oncogenesis.

[200] Cox TR (2021). The matrix in cancer. Nat Rev Cancer.

[201] Vandenbroucke RE, Libert C (2014). Is there new hope for therapeutic matrix metalloproteinase inhibition?. Nat Rev Drug Discov.

[202] Liu J, Chen T, Li S, Liu W, Wang P, Shang G (2022). Targeting matrix metalloproteinases by E3 ubiquitin ligases as a way to regulate the tumor microenvironment for cancer therapy. Semin Cancer Biol.

[203] Lee H, Tsygankov AY (2010). c-Cbl regulates glioma invasion through matrix metalloproteinase 2. J Cell Biochem.

[204] Chen L, Yuan R, Wen C, Liu T, Feng Q, Deng X (2021). E3 ubiquitin ligase UBR5 promotes pancreatic cancer growth and aerobic glycolysis by downregulating FBP1 via destabilization of C/EBPα. Oncogene.

[205] Shangguan X, He J, Ma Z, Zhang W, Ji Y, Shen K (2021). SUMOylation controls the binding of hexokinase 2 to mitochondria and protects against prostate cancer tumorigenesis. Nat Commun.

[206] Lin HP, Cheng ZL, He RY, Song L, Tian MX, Zhou LS (2016). Destabilization of fatty acid synthase by acetylation inhibits de novo lipogenesis and tumor cell growth. Cancer Res.

[207] Jin MZ, Jin WL (2020). The updated landscape of tumor microenvironment and drug repurposing. Signal Transduct Target Ther.

[208] Jiang Y, Zhang H, Wang J, Liu Y, Luo T, Hua H (2022). Targeting extracellular matrix stiffness and mechanotransducers to improve cancer therapy. J Hematol Oncol.

[209] Liu F, Qin L, Liao Z, Song J, Yuan C, Liu Y (2020). Microenvironment characterization and multi-omics signatures related to prognosis and immunotherapy response of hepatocellular carcinoma. Exp Hematol Oncol.

[210] Roma-Rodrigues C, Mendes R, Baptista PV, Fernandes AR (2019). Targeting tumor microenvironment for cancer therapy. Int J Mol Sci.

[211] Haslam A, Prasad V (2019). Estimation of the percentage of US patients with cancer who are eligible for and respond to checkpoint inhibitor immunotherapy drugs. JAMA Netw Open.

[212] Yi M, Jiao D, Qin S, Chu Q, Wu K, Li A (2019). Synergistic effect of immune checkpoint blockade and anti-angiogenesis in cancer treatment. Mol Cancer.

[213] Xiao N, Zhu X, Li K, Chen Y, Liu X, Xu B (2021). Blocking siglec-10(hi) tumor-associated macrophages improves anti-tumor immunity and enhances immunotherapy for hepatocellular carcinoma. Exp Hematol Oncol.

[214] Salami J, Crews CM (2017). Waste disposal-An attractive strategy for cancer therapy. Science.

[215] Dale B, Cheng M, Park KS, Kaniskan H, Xiong Y, Jin J (2021). Advancing targeted protein degradation for cancer therapy. Nat Rev Cancer.

[216] Sakamoto KM, Kim KB, Kumagai A, Mercurio F, Crews CM, Deshaies RJ (2001). Protacs: chimeric molecules that target proteins to the Skp1-Cullin-F box complex for ubiquitination and degradation. Proc Natl Acad Sci U S A.

[217] Li H, Dong J, Cai M, Xu Z, Cheng XD, Qin JJ (2021). Protein degradation technology: a strategic paradigm shift in drug discovery. J Hematol Oncol.

[218] Neklesa TK, Winkler JD, Crews CM (2017). Targeted protein degradation by PROTACs. Pharmacol Ther.

[219] Nalawansha DA, Crews CM (2020). PROTACs: an emerging therapeutic modality in precision medicine. Cell Chem Biol.

[220] Liu J, Ma J, Liu Y, Xia J, Li Y, Wang ZP (2020). PROTACs: a novel strategy for cancer therapy. Semin Cancer Biol.

[221] Mullard A (2019). Arvinas’s PROTACs pass first safety and PK analysis. Nat Rev Drug Discov.

[222] Mullard A (2020). Targeted degraders clear first safety hurdles. Nat Rev Drug Discov.

[223] Mullard A (2019). First targeted protein degrader hits the clinic. Nat Rev Drug Discov.

[224] Liu J, Peng Y, Inuzuka H, Wei W (2022). Targeting micro-environmental pathways by PROTACs as a therapeutic strategy. Semin Cancer Biol.

[225] Li X, Yao Y, Wu F, Song Y (2022). A proteolysis-targeting chimera molecule selectively degrades ENL and inhibits malignant gene expression and tumor growth. J Hematol Oncol.

[226] Neklesa TK, Tae HS, Schneekloth AR, Stulberg MJ, Corson TW, Sundberg TB (2011). Small-molecule hydrophobic tagging-induced degradation of HaloTag fusion proteins. Nat Chem Biol.

[227] McClellan AJ, Tam S, Kaganovich D, Frydman J (2005). Protein quality control: chaperones culling corrupt conformations. Nat Cell Biol.

[228] Chamberlain PP, Hamann LG (2019). Development of targeted protein degradation therapeutics. Nat Chem Biol.

[229] Ito T, Ando H, Suzuki T, Ogura T, Hotta K, Imamura Y (2010). Identification of a primary target of thalidomide teratogenicity. Science.

[230] Lu G, Middleton RE, Sun H, Naniong M, Ott CJ, Mitsiades CS (2014). The myeloma drug lenalidomide promotes the cereblon-dependent destruction of Ikaros proteins. Science.

[231] Krönke J, Udeshi ND, Narla A, Grauman P, Hurst SN, McConkey M (2014). Lenalidomide causes selective degradation of IKZF1 and IKZF3 in multiple myeloma cells. Science.

[232] Banik SM, Pedram K, Wisnovsky S, Ahn G, Riley NM, Bertozzi CR (2020). Lysosome-targeting chimaeras for degradation of extracellular proteins. Nature.

[233] Caianiello DF, Zhang M, Ray JD, Howell RA, Swartzel JC, Branham EMJ (2021). Bifunctional small molecules that mediate the degradation of extracellular proteins. Nat Chem Biol.

